# On the lack of a universal pattern associated with mammalian domestication: differences in skull growth trajectories across phylogeny

**DOI:** 10.1098/rsos.170876

**Published:** 2017-10-25

**Authors:** Marcelo R. Sánchez-Villagra, Valentina Segura, Madeleine Geiger, Laura Heck, Kristof Veitschegger, David Flores

**Affiliations:** 1 Palaeontological Institute and Museum, University of Zurich, Karl-Schmid-Strasse 4, 8006 Zurich, Switzerland; 2 Unidad Ejecutora Lillo, Consejo Nacional de Investigaciones Científicas y Técnicas-Fundación Miguel Lillo, Argentina; 3 Department of Zoology, University of Cambridge, Downing Street, Cambridge, CB2 3EJ, UK

**Keywords:** ontogeny, development, dog, cat, horse, modularity

## Abstract

As shown in a taxonomically broad study, domestication modifies postnatal growth. Skull shape across 1128 individuals was characterized by 14 linear measurements, comparing 13 pairs of wild versus domesticated forms. Among wild forms, the boar, the rabbit and the wolf have the highest proportion of allometric growth, explaining in part the great morphological diversity of the domesticated forms of these species. Wild forms exhibit more isometric growth than their domesticated counterparts. Multivariate comparisons show that dogs and llamas exhibit the greatest amount of differences in trajectories with their wild counterparts. The least amount is recorded in the pig–boar, and camel and horse pairs. Bivariate analyses reveal that most domesticated forms have growth trajectories different from their respective wild counterparts with regard to the slopes. In pigs and camels slopes are shared and intercepts are different. There is a trajectory extension in most domesticated herbivores and the contrary pattern in carnivorous forms. However, there is no single, universal and global pattern of paedomorphosis or any other kind of heterochrony behind the morphological diversification that accompanies domestication.

## 1. Introduction

Genomic and archeological studies have helped to establish with great certainty the wild species from which domesticated mammals originated [1], and many of the genetic bases of some of the traits that arise with domestication and selective breeding have been discovered [2–4]. Phenomic studies are lagging behind, although these would be fundamental to understand what at the end fascinates us today as it fascinated Darwin as well: the morphological diversity (disparity) in domesticated forms. Here an ontogenetic perspective is fundamental, given its centrality to understand the evolution of form [5].

The power of selective breeding to produce morphological diversity, as best exemplified by the case of dogs, is uncontested [6–9]. But are there intrinsic aspects in the biology of the dog that make it a particularly plastic species? How do dogs compare with other domesticated forms? To answer these questions, we need to compare the fundamental pattern of differentiation, of development, that characterizes the species to be compared.

In the case of mammals, much of the differentiation among species occurs during the postnatal period, although many species-specific features are already established at birth [10,11]. The most widely used marker of morphological diversity is the skull, given its complexity in form (shape and size) and embryological origin (mesodermal and neural crest; pharyngeal arches, dermatocranium and endocranium; [12]), and its relation to organs such as the brain, to sensory organs and to feeding function. Studies of skulls benefit from the fact the museum collections keep specimens that are available for study, in some cases of populations no longer existing in the wild.

**Figure 1.**
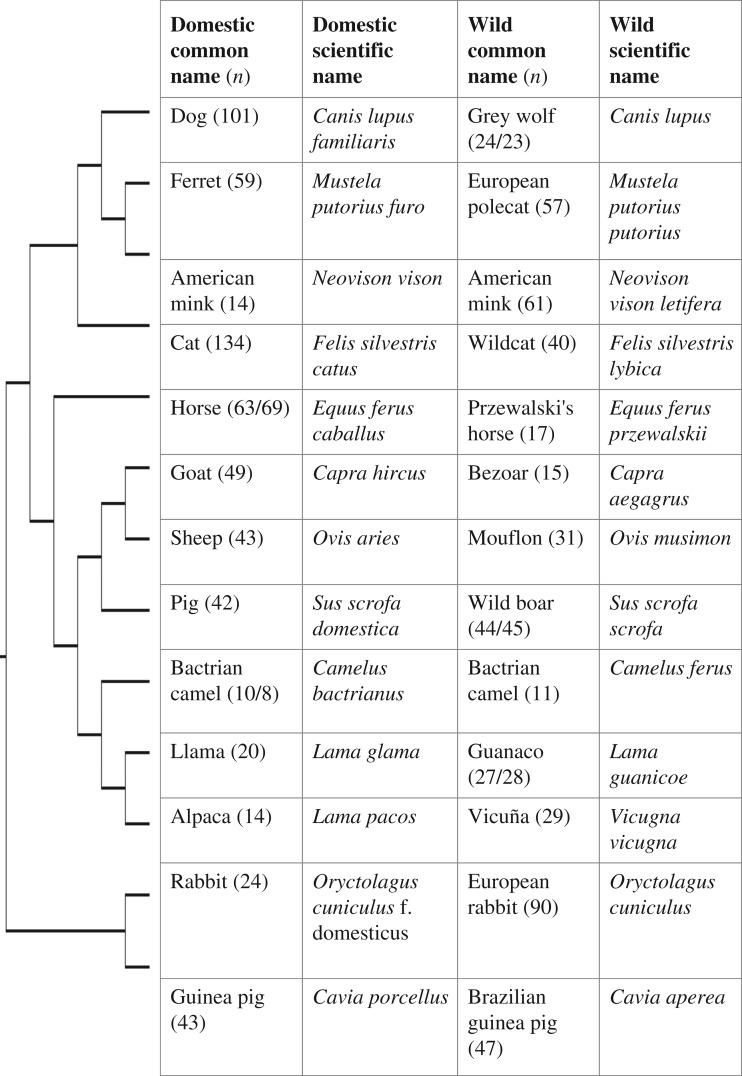
Pairs of domesticated versus wild forms investigated in this work. The number of specimens investigated is indicated in brackets, for both multivariate and bivariate analyses; in case they differ they are listed first and second, respectively. Names of taxa followed most common current use (e.g. [13]).The names of the species provided fits current use, although some of it is not universal and is indeed controversial [14].

We present a comprehensive examination of skull growth trajectories in 13 wild versus domesticated forms of mammals, including members of all major clades in which domestication has occurred (figure 1). By using a similar method and measurements protocol, we can for the first time investigate the similarities and differences in the changes produced by domestication.

Although a synthetic and comparable quantification of morphospace occupation among domesticated mammals is still in its infancy, previous works [8,15] have stated what newer methods are likely to confirm: some species have become more morphologically diverse than others [9]. Dogs and pigs, for example, are more morphologically diverse than cats and horses [16]. What is behind these patterns? Our study addresses the potential effects of growth patterns in the diversification associated with domestication, with a dataset that leaves no ambiguity about comparisons across species and that is broad in taxonomic scope.

## 2. Material and methods

**Figure 2.**
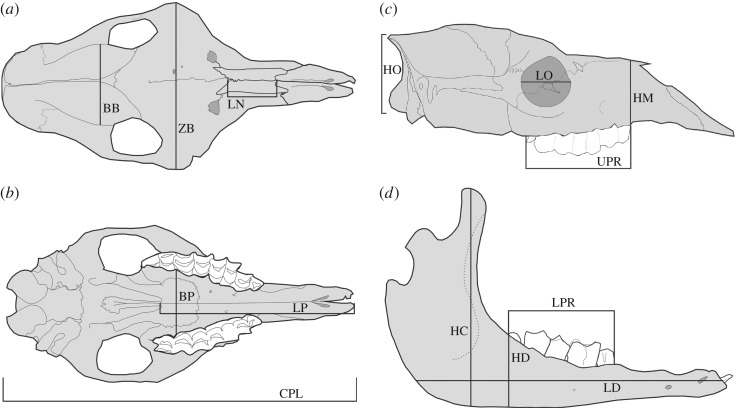
Measurements of the skull in dorsal (*a*), ventral (*b*) and lateral (*c*) views and the mandible (*d*). BB, breadth of the braincase; BP, breadth of palate; CPL, condylo-premaxillary length; HC, height of the coronoid process; HD, height of the dentary; HM, height of the muzzle; HO, height of occipital plate; LD, length of the dentary; LN, length of the nasals; LO, length of the orbit; LP, length of palate; LPR, length of lower post-canine row; UPR, length of upper post-canine or of molariform tooth row; ZB, zygomatic breadth. See electronic supplementary material, 1, for details.

Our study of extensive growth series (figure 1) is based on 1128 specimens deposited in 15 institutions in eight countries (electronic supplementary material, 1). In each species we aimed at having individuals evenly distributed in size and representing as much as possible of the postnatal growth trajectory. For all species we considered young specimens with deciduous teeth or incomplete adult dentition, with the smallest specimens being 50% the size or less than the larger specimens. We took 14 skull measurements that serve to optimize the characterization of the skull shape for a broad sample of species (figure 2; electronic supplementary material, 1).

In many of the studied species, domestication has been estimated to have started between 10 000 and 5000 years ago, dogs having been domesticated earlier and rabbits and minks in much more recent times [1]. It would be ideal to have access to growth series of the original wild forms, at the time of the beginning of their domestication, but this is obviously impossible. We then sampled the best approximation to it: modern wild individuals when possible from the geographic area of origin of domestication (electronic supplementary material, 1). We sampled domesticated individuals from populations that do not represent specialized breeds (e.g. short snouted varieties), but instead a more generalized domesticated form, thus the best approximation to the fundamental aspect of domestication and not the result of intense selective breeding for some specific trait.

For ontogenetic pattern comparisons, we performed both multivariate and bivariate analyses. The former considers size as a latent variable affecting all measured variables simultaneously and is thus more realistic, whereas bivariate approach is suitable for statistical comparison of slopes and intercepts of regressions for wild versus domestic forms.

In our bivariate and multivariate approach, we pooled all ages together assuming a uniform growth rate, until reaching the final adult size (i.e. offset of ontogenetic regression). The mode of growth of some carnivorans (e.g. pinnipeds), which exhibit specialized social behaviour, includes a second spurt in adult males, as reflected in significant differences in young and adult males trajectories linked to extreme sexual dimorphism (e.g. [17]). Such condition was not observed in previous analyses in terrestrial carnivorans [18,19], even considering large felids [19] and polygamous herbivores [20]. Such evidence assumes non-significant differences in young and adult growth trajectories. The same can be assumed with respect to sexual dimorphism, in which several reports suggest the same growth trajectories for both sexes in terrestrial carnivores and herbivores (e.g. [18,20]).

### 2.1. Multivariate approach

The multivariate approach used (e.g. [21]) evaluated the first (unit-scaled) eigenvector of a principal component analysis (PCA) based on a variance–covariance matrix of log10 transformed data for all variables and for each taxon [22]. For a given variable, allometry is the statistical deviation of its corresponding eigenvector element from the hypothetical isometric value (i.e. if the global growth pattern is size invariant), which is calculated as 1/*p*^0.5^ with *p* equal to the number of variables. In order to generate confidence intervals for each of the empirically derived first-eigenvector elements, statistical deviation from isometry was estimated using the application of jackknife [23]. The generated confidence interval may be inclusive of the isometric value and, therefore, indistinguishable from isometry, or it may exclude such value and, therefore, be considered significantly allometric (i.e. positive or negative, with a higher or lower rate of change for the specific variable when compared with overall growth). From the collection of *n* pseudovalues obtained from a resampling strategy, in which one specimen by PCA round is eliminated in the sample [23], mean and standard deviation were calculated for each element corresponding to one skull variable. The mean represents the raw jackknife estimate of the multivariate allometry coefficient for that variable. The difference between this estimate and the actual value from the complete sample is a measure of bias [24]. Confidence intervals may be severely influenced by extreme pseudovalues (those obtained by the resampling just described) and trimming the *m* largest and the *m* smallest values decreased the standard deviations and allowed for better allometric estimations [24]. We report untrimmed as well as (*m* = 1) trimmed calculations of confidence intervals, opting for the results with either lower average standard deviation or bias. The statistical analyses (PCA + jackknife resampling) were programmed in R [25]; the script is available on request.

The multivariate coefficients of allometry are expressed as confidence intervals. They can show different signs in their allometric trend (positive and negative allometry) and no intersection in their extreme values, showing an absolute value in their difference defined as the distance between the higher and lower limits of both intervals. Similarly, the two compared confidence intervals derived from multivariate analyses can show the same allometric trend, but with no overlap in values. In other cases, both compared intervals can show intersection in their values, and thus no absolute differences, but one of them including the hypothetical value of isometry (1/*p*^0.5^, 0.267 for this study), showing in consequence different sign in their allometric trends. In other cases, both intervals can share the same allometric sign with intersection of their values.

In order to quantify the changes between wild and domesticated forms (confidence intervals), we added the individual change of all variables as an ‘added change’ for each comparison between forms, and also the ‘added change’ for each cranial variable across our 13 comparisons (table 1).

**Table 1.** Comparisons of growth trajectories between domesticated and wild forms, based on multivariate analysis of 14 linear measurements of the skull and dentary bone. In each cell we report the difference between the confidence intervals of the rate of growth (i.e. allometric coefficients) obtained for each variable and for each species/form (see Material and methods). The cells in dark grey show the greatest amount of change, those with the value in 0 in light grey indicate the cases in which the difference in the growth trend exists, although the confidence intervals (CI) overlap (intersect). We treated the growth trajectory (allometric) values as continuous characters and we subtracted the amount of change between each wild–domestic pair. For CI choice based on trimmed or untrimmed sample of pseudovalues see Material and methods and electronic supplementary material, 3. The question marks result from the lack of data on the dentaries of goats. We added the individual values for all variables as ‘added change’ for each species/form, and also for the same variable for all species. Abbreviations as in figure 2.

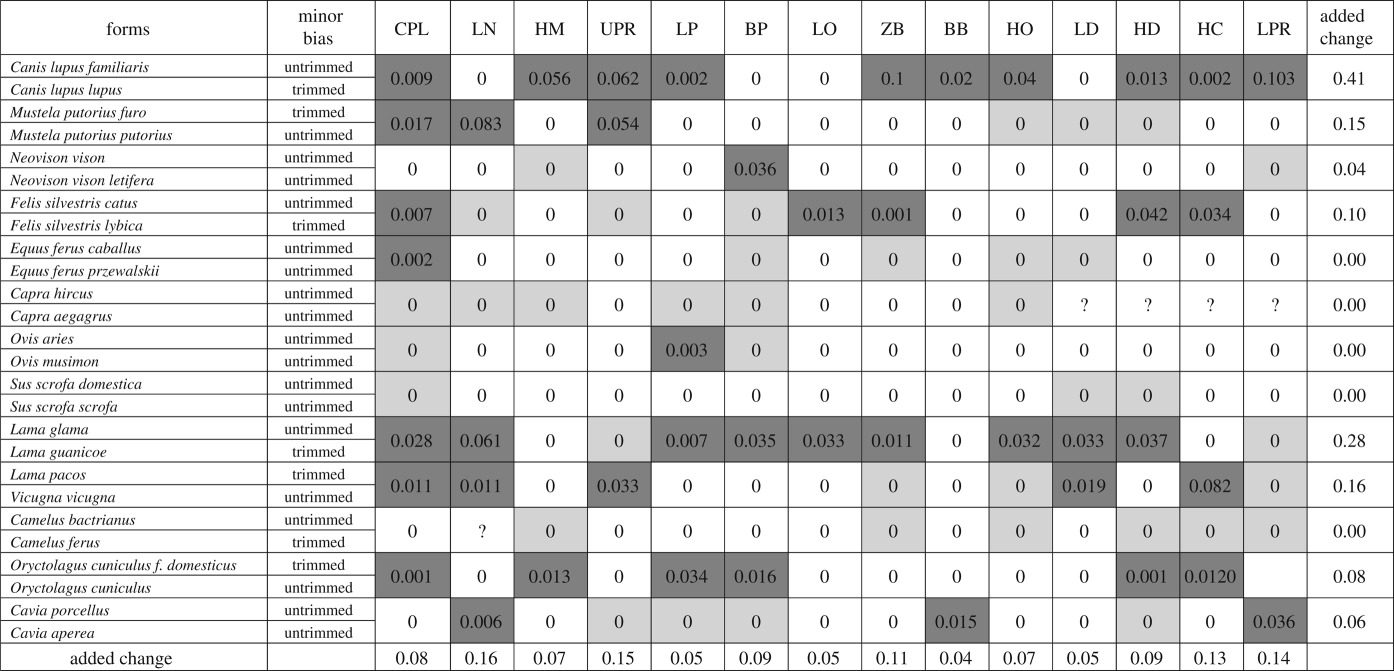

### 2.2. Bivariate approach

We compared proportions of different cranial parts across species. Ontogeny is expressed as a linear regression, in which the time frame is implicitly incorporated (size proxy), in order to describe relative modifications as the individuals grow and examine potential heterochronic processes. We interpreted coefficients of allometry as growth rates (e.g. [26,27]). Overall size was estimated as the geometric mean [28], which ensures the isometric condition of the independent variable. The relation of each variable to overall size was examined using the standard allometric equation derived from a power growth function converted to its (base10) logarithm [29].

We performed *F*-tests with the null coefficient set at 1.0 to assess deviations from isometry [27,30], after corroborating that the independent variable (i.e. geometric mean) was normally distributed (Shapiro–Wilk test, electronic supplementary material, 1). We examined differences in ontogenetic regressions comparing slopes and intercepts of the linear trajectories (table 2). Significance level was set to *p* = 0.0036; that *p*-value representing the usual 5% alpha level divided by the number of statistical tests (14 regressions) performed over the same sample units (Bonferroni correction; [31]). For those regressions that exhibit the same slopes and intercepts, we evaluated the existence of ‘shifts’, meaning any significant extensions or truncations of the domestic trajectory with respect to the wild one. For such comparisons we applied standardized major axis regression (SMA; [27]) and followed Warton *et al*. [32] in order to test the common SMA slope using a *χ*^2^ distribution [30]. In those cases where domesticated and wild forms shared a common slope, we compared the significance of the common *y*-intercepts using the Wald test (as described in [32]). All regression coefficients, statistical parameters, and tests were performed using the smatr package in R [30]. In our comparison of growth trajectories between domesticated and wild forms, we considered the wild form as the ancestor of the domesticated one, from which a heterochronic pattern may derive or not in the domesticated form. Following Reilly *et al*. [33], peramorphosis (extended development) is produced by an increase in rate (acceleration, larger slope in the domesticated than in the wild form), a later offset time (hypermorphosis, trajectory extension in the domesticated form), or an earlier onset time (pre-displacement of the trajectory in the domesticated form). Conversely, paedomorphosis (results in traits produced by truncated development) is produced by a slower rate (deceleration, small slope), an earlier offset time (hypomorphosis, trajectory truncation in the domesticated form) or a later onset time (post-displacement of the trajectory in the domesticated form). Changes in the intercept would either indicate neomorphy or—together with changes in slope and/or onset and offset time—be indicative of heterochronic changes.

**Table 2.** Summary of allometric trends in the 13 wild and domesticated forms for 14 skull variables investigated. The used symbols are: ‘‘+’’ (accelerated with respect to overall size or positive allometric), ‘‘−’’ (decelerated with respect to overall size or negative allometric), ‘‘=’’ (isometric). U, untrimmed sample of pseudovalues; T, trimmed sample of pseudovalues. Abbreviations as in figure 2.

forms	minor bias	CPL	LN	HM	URP	LP	BP	LO	ZB	BB	HO	LD	HD	HC	LPR	totals
*Canis lupus familiaris*	U	+	+	−	+	=	−	−	−	−	−	+	−	+	+	6P,1I,7N
*Canis lupus lupus*	T	−	+	+	=	−	−	−	+	−	+	+	=	+	−	6P,2I,6N
*Mustela putorius furo*	T	+	−	=	+	+	−	−	=	−	=	+	−	+	=	5P,4I,5N
*Mustela putorius putorius*	U	=	+	=	=	+	−	−	=	−	−	=	=	+	=	3P,7I,4N
*Neovison vison*	U	−	=	+	−	=	−	−	=	−	=	=	+	=	−	2P,6I,6N
*Neovison vison letifera*	U	−	=	=	−	=	−	−	=	−	=	=	+	+	=	2P,7I,6N
*Felis silvestris catus*	U	=	+	=	−	=	+	=	+	−	−	+	+	+	−	6P,4I,4N
*Felis silvestris lybica*	T	−	=	=	=	=	=	−	=	−	−	+	+	+	−	3P,6I,5N
*Equus ferus caballus*	U	+	+	+	=	+	−	−	−	−	=	+	=	+	=	6P,4I,4N
*Equus ferus przewalskii*	U	=	+	+	=	+	=	−	=	−	−	=	=	+	=	4P,7I,3N
*Capra hircus*	U	+	+	+	=	+	−	−	−	−	−	?	?	?	?	4P,1I,5N
*Capra aegagrus*	U	=	=	=	=	=	=	−	=	−	=	?	?	?	?	8I,2N
*Ovis aries*	U	+	+	+	=	+	−	−	−	−	−	=	−	+	+	6P,2I,6N
*Ovis musimon*	U	=	+	+	=	=	=	−	−	−	−	=	−	+	+	4P,5I,5N
*Sus scrofa domestica*	U	=	+	−	+	+	−	−	−	−	−	=	=	+	+	5P,3I,6N
*Sus scrofa scrofa*	U	+	+	−	+	+	−	−	−	−	−	+	+	+	+	8P,0I,6N
*Lama glama*	U	+	+	=	=	+	−	−	−	−	=	+	−	+	−	5P,3I,6N
*Lama guanicoe*	T	−	+	=	−	+	=	−	−	−	−	−	=	+	=	3P,4I,7N
*Lama pacos*	T	−	+	=	−	=	−	−	−	−	−	−	−	−	−	1P,2I,11N
*Vicugna vicugna*	U	=	+	=	=	=	−	−	=	−	=	=	−	=	=	1P,9I,4N
*Camelus bactrianus*	U	=	?	+	=	+	−	−	=	−	=	=	=	=	=	2P,8I,3N
*Camelus ferus*	T	=	?	=	=	+	−	−	+	−	−	=	−	+	+	4P,4I,5N
*Oryctolagus c. f. domesticus*	T	+	+	+	−	+	−	−	−	−	−	+	+	+	−	7P,7N
*Oryctolagus cuniculus*	U	=	+	−	−	+	−	−	−	−	−	+	+	+	−	5P,1I,8N
*Cavia porcellus*	U	=	=	=	−	=	+	−	=	−	−	=	=	=	=	1P,9I,4N
*Cavia aperea*	U	=	+	=	=	+	=	−	=	−	−	=	−	=	−	2P,7I,5N

## 3. Results

Based on the multivariate comparison of growth trajectories between wild and domesticated forms, the following patterns emerge (table 1). Wolves–dogs and llamas–guanacos are the pairs that exhibit the greatest amount of added change (0.407 and 0.277, respectively). In fact, the dog is more diverging from wolf than are cats and horses from their wild counterparts (they exhibit values of 0.097 and 0.002, respectively). The species with the least amount of differences wild versus domesticated are the pig, camel and goat.

Considering all 13 comparisons wild versus domesticated (electronic supplementary material, 2), there are clear differences in the amount of change of variables, expanding one order of magnitude (table 1). The variables showing lower added change values are the breadth of the braincase, the length of the orbit and the length of the palate (0.035, 0.0460 and 0.0461, respectively). The length of the nasals is the variable showing the greatest amount of added change (0.161). The variables correlated with the trophic apparatus, upper and lower tooth rows, the height of the coronoid process and zygomatic width, have the following added change values: 0.149, 0.139, 0.130 and 0.112, respectively.

In general, the wild forms exhibit more isometric growth than the domesticated counterparts, which show more allometric growth (table 2). The number of measurements with isometric growth in wild and domesticated form is the following: wolf–dog: 2 : 1, polecat–ferret: 7 : 4, wild–domesticated mink: 7 : 6, wild–domesticated cat: 6 : 4, Przewalski's horse–horse: 7 : 4, bezoar–goat: 8 : 1, mouflon–sheep: 5 : 2, guanaco–llama: 4 : 3, vicuña–alpaca: 9 : 2, wild–domesticated rabbit: 1 : 0. In only three cases of 13 is there more isometric growth in the domesticated form, with the following numbers: boar–pig: 0 : 3; wild–domesticated Bactrian camel: 4 : 8; wild–domesticated guinea pig: 7 : 9 (table 2).

**Figure 3.**
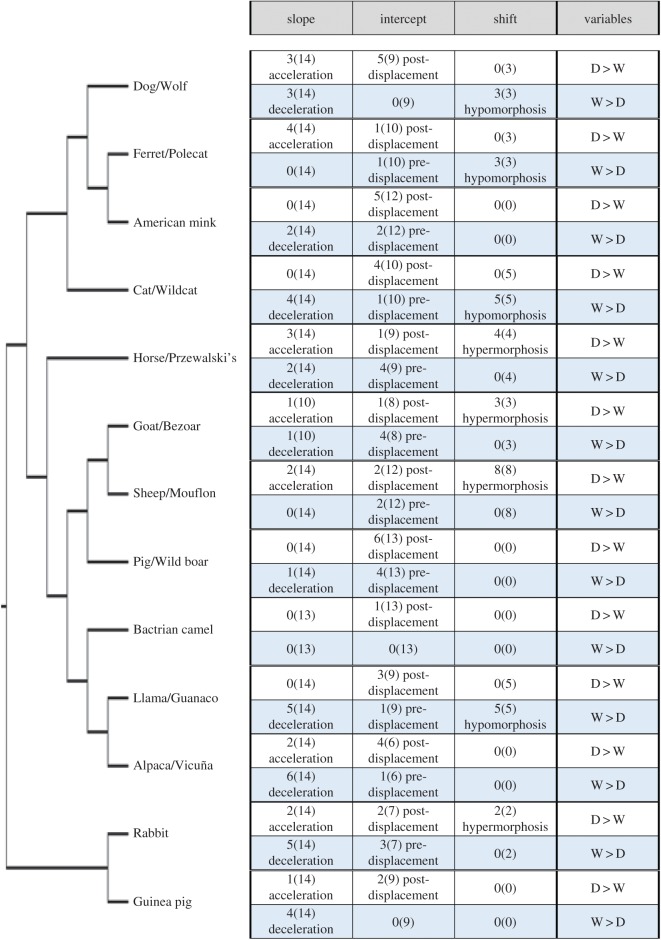
Summary of results of the bivariate analysis of ontogenetic trajectories of 14 skull variables in the investigated 13 wild and domesticated forms. The symbols indicate the cases in which domesticates (D) were larger than wild (W) forms (or vice versa) out of the 14 relations between size (with geometric mean as a proxy for it) and the variable in question. For those trajectories that exhibit the same slopes and intercepts, we evaluated which form, if any, exhibits significant extensions of the trajectory with respect to the other (shift). The terminology of heterochrony follows Reilly *et al*. [33]. See electronic supplementary information for a detailed list of the changes for the individual variables (figure 2).

Based on the comparison of growth trajectories between domesticated and wild forms (figure 3), we found that the total number of heterochronic events was high (14) in dog–wolf, cat, horse–Przewalski's horse, sheep, llama and rabbit, whereas in camel and guinea pig the number was low (1 and 7, respectively).

Most domesticated forms have growth trajectories different from their respective wild counterparts (figure 3, electronic supplementary material, 4, 5) with regard to the slopes (e.g. New World camelids, rabbits, dogs, cats, horses and guinea pigs). By contrast, in pigs and camels the slopes are mostly shared, with differences recorded in the intercepts (figure 3).

Common heterochronic patterns across comparisons between domesticated and wild regressions exist—here we refer to changes in slope and intercept, and when these are similar, to ‘shifts’ (figure 3). Except in the case of the mink, where there is no shift difference between both forms, in carnivorans the domestic forms show a shorter growth trajectory in contrast to the wild forms (figure 3; electronic supplementary material, 3, 4). This change we consider a pattern of hypomorphosis. By contrast, in other species, which are herbivores, we recorded an extension of the growth trajectory in most domesticated forms (horse, goat, sheep and rabbit) corresponding to hypermorphosis as the heterochronic pattern (figure 3). In others, such as pig, Bactrian camel, guinea pig and vicuña, there is no difference with the wild form in the extension of the growth trajectory. Just in the guanaco–llama case, the shift is higher in the wild form. In most comparisons between wild and domesticated forms, the neurocranial variables showed significant differences in their slopes, intercepts or shift. Our results based on multivariate and bivariate approaches are in general in agreement. Those multivariate coefficients of allometry that show differences in their confidence intervals (i.e. no overlap, table 1) also exhibit significant differences in the slope of ontogenetic regressions (figure 3; electronic supplementary material, 3, 4).

Bivariate regressions show in most cases high values of correlation of cranial variables with the geometric mean, with exceptions (electronic supplementary material, 5). In most cases, the lower correlation corresponds to the breadth of the braincase (e.g. wolf 0.0014; vicuña 0.1301; guanaco 0.1655; domesticated guinea pig 0.2401; wild cat 0.2507, polecat 0.2640, horse 0.3670; alpaca 0.3723; sheep 0.4078; mouflon 0.5567; wild mink 0.5631; bezoar 0.5839).

## 4. Discussion

The investigated species have different trajectory patterns, and the changes in trajectories recorded in wild versus domesticated forms are not equal across mammals. There is no single, universal pattern of heterochrony associated with domestication. The recognition of some common features across species as part of the ‘domestication syndrome’ [34] should not lead to the assumption of commonality in domestication. In fact, the phenotypic patterns of change reported for canids are not universal across mammals [5]. The same is true for ontogeny.

The differences across species in the amount of change in skull proportions with growth (table 1) probably constitute one of the most important factors affecting the amount of morphological diversity a species can attain through selective breeding—their evolvability [35]. Non-isometric (allometric) growth of species (table 2) has been considered as an indicator that the domestication process of that species may have more potential for generating morphological disparity [16,36]. This is because with even minor changes in size, allometric growth produces different proportions and in some cases thus disparity. In contrast, isometric growth implies that two individuals of different size basically look alike. For instance, for wolves, the large amount of allometric change—characteristic of canids [36]—suggest an intrinsic propensity for change—given its allometric growth. In the dog, as in the wolf, most variables also exhibit the allometric growth pattern (table 2). The isometric growth of cats has been claimed to explain at least in part the relative conservatism in this species when compared with dogs [16,36–38]. In our study, we found that with domestication there is more allometric growth (table 2). For example, wild cats exhibit more isometric growth than domesticated ones (table 2).

Insights are also provided by comparing across species the bivariate trajectories. These are slightly more conserved in cats than in dogs (figure 3). In cats, wild and domesticated forms share the same slopes for most variables, with some differences mainly in the intercepts (i.e. pre- and post-displacement) and shifts (i.e. trajectory extension), whereas in the case of dogs more variables showed different slopes (figure 3). Indeed, the added change between wild and domesticated dogs in multivariate analyses is higher than in all other pairs of wild versus domesticated analysed (table 1).

The boar, the wild rabbit and the wolf are in their wild forms the species with the highest proportion of allometric growth, with just 0, 1 and 2 isometric relations, respectively (table 2). The disparity in the domesticated forms of these three cases is large and much larger than in other cases of domestication [16]—thus supporting the link between allometric growth and potential to become morphological diverse. Dogs and pigs are reportedly more morphologically diverse than cats and horses [16,39]. The skull disparity among domesticated rabbit populations that has evolved since the domestication in the Middle Ages [40,41] has not been quantified, but the external phenotype hints that this could be significant [42].

By virtue of having compared the most generalized domesticated form to each wild form of a species, we can approximate the changes having taken place in the initial phases of domestication [43], which concerns mostly the attainment of tameness and the skull changes and other features associated with it [34]. As such, we are not concerned here with the generation of the extremes of morphospace occupation that result from intense selection for a trait or the accentuation of particular features in special breeds. Thus, the amount of change we record in allometric patterns between wild and domesticated forms in our study reflect more an intrinsic aspect of the species growth as opposed to the effects of selection.

### 4.1. Developmental repatterning

Heterochrony or changes in developmental timing have been proposed as common or at least relevant in understanding changes in domestication [39]. This is confirmed for the skull, but the pattern is neither global nor uniform across species. Our results demonstrate different heterochronic patterns describe the differences between wild and domestic forms for different regions of the skull (figure 3). Even considering specifically those variables related with the neurocranium (i.e. orbit length, breadth of the braincase and occipital height), whose negative allometry is conservative across species, the highly frequent differences in trajectory between wild and domestic forms implies diverse heterochronic processes (i.e. differences in slope, intercepts and trajectory extension).

That different parts of the skull change differently [44] was to be expected given the modularity of the skull [45,46], which shows that if developmental repatterning occurs in the form of heterochrony [47], the resulting pattern is not global. This had been found, for example, when comparing wild versus domesticated guinea pigs [48] and wild boar versus domesticated pigs [49]. Our study has been about postnatal changes, which are the ones classically studied in comparative studies of growth. Based on previous works, we know that in order to understand how ontogeny varies in evolution, prenatal changes can also be important [10,50]. This has been shown for wild versus domestics in the wolf/dog case [51], and is likely to apply to other species as well. Geiger *et al*. [51] showed that the skull shape of adult dogs is both neomorphic and paedomorphic. Dog skulls show unique features already shortly after birth, whereas at any given age, juvenile dogs exhibit skull shapes that resemble those of younger wolves.

### 4.2. Skull modules

There is conservatism in many aspects of postnatal growth trajectories, i.e. universal patterns of allometric or isometric growth for some of the skull parts in the wild and domesticated forms for the different species. Based on studies of diverse mammalian species and clades, it is well established that during growth the neurocranial components of the skull, mostly related to the brain and sensory organs, scale negatively whereas the splanchnocranial components, related to the masticatory apparatus scale positively with size (e.g. [21,52–55]). Not surprisingly, this trend was found for all species studied (table 2). The measured skull variables related to the splanchnocranium exhibit in general more accumulated changes than the neurocranial ones (such as breadth of the braincase and occipital height; tables 1 and 2), with the exception of the height of the coronoid process and breadth of the palate. Height of the coronoid process shows positive allometry in almost all ontogenetic series analysed, suggesting an accelerated postnatal growth related with the insertion of masticatory muscles. On the other hand, the generalized negative allometry recorded in the breadth of the palate indicates a wide palate from early stages of postnatal development, which serves as a platform for the tongue during the complex process of suction during lactation in mammals (e.g. [26].

Recently, the subject of modularity as it relates to dog domestication has been examined [56], and recent work on pig skull growth looked at modules of the skull and their differential growth [49]. The subject of modularity as affected by domestication and its relation to diversification is in its infancy, including the ontogenetic perspective to the subject, in spite of its great relevance to understand the mechanistic bases of integration [47,57].

## 5. Conclusion

We demonstrate that domestication has influenced postnatal growth trajectories. However, there is no ‘domestication syndrome’ for ontogeny, as there is no single, universal pattern that accompanies domestication. The discovered patterns exemplify the complex nature of evolutionary changes in the skull during domestication, and show that these changes cannot be simply described as cases of either neomorphy or heterochrony [58].

The conservatism in many aspects of postnatal growth trajectories means that many of the differences among species may exist already around birth, and that the study of prenatal ontogeny of the skull is also important to understand the paths of differentiation across species [10,11]. We hypothesize that domestication has influenced prenatal growth patterns, a case of ‘developmental penetrance’ of evolutionary change [59].

## Supplementary Material

Supplementary Information 1

## Supplementary Material

Supplementary Information 2

## Supplementary Material

Supplementary Information 3

## Supplementary Material

Supplementary Information 4

## Supplementary Material

Supplementary Information 5

## Supplementary Material

Supplementary Information 6

## Data accessibility

See electronic supplementary material.
