# Supplementary material for: On the lack of a universal pattern associated with mammalian domestication: differences in skull growth trajectories across phylogeny
Source: R Soc Open Sci. 2017 Oct 25;4(10):170876. doi: 10.1098/rsos.170876 (PMC5666271; doi:10.1098/rsos.170876)
Supplement: Supplementary Information 1 [file rsos170876supp1.docx]

**Supplementary Information**

**On the lack of a universal pattern associated with domestication - differences in skull growth trajectories across mammals**

Marcelo R. Sánchez-Villagra*, Valentina Segura, Madeleine Geiger, Laura Heck, Kristof Veitschegger, David Flores

**Supplementary Information 1.** Description of Institutions/curators, choice of species and populations sampled, range of size and test of normality and

measurements

Contents of Supplementary Information 1.

Supplementary Information 1 A. List of Institutions/curators from which specimens were studied

Supplementary Information 1 B. On the choice of species and populations sampled for the wild / domesticated comparisons

Supplementary information 1 C. Range of size (geometric mean) and test of normality (Shapiro-Wilk test) in domestic and wild forms.

Supplementary information 1 D. Description of measurements

**Supplementary Information 1 A**. List of Institutions/curators from which specimens were studied

USA: American Museum of Natural History, New York (ANMH), Eileen Westwig.

Smithsonian Institution National Museum of Natural History, Washington DC (NMNH), Darrin Lunde and Esther M. Langan.

Switzerland: Kynologische Sammlung der Albert-Heim-Stiftung, Naturhistorisches Museum Bern, (NMBE), Marc Nussbaumer.

Naturhistorisches Museum Basel (NHMB), Loic Costeur.

Palaeontologisches Institut und Museum, University of Zurich (PIMUZ), Gabriel Aguirre-Fernández

Argentina: Museo de la Plata, Argentina (MLP), A. Itatí Olivares, Diego Verzi.

Museo Argentino de Ciencias Naturales Bernardino Rivadavia, Buenos Aires (MACN), Pablo Teta, Guillermo Cassini and Sergio Lucero.

Germany: Museum für Naturkunde, Berlin (MfN), Christiane Funk.

Senckenberg Museum, Frankfurt (SM), Irina Ruf and Katrin Krohmann.

Staatssammlung für Anthropologie und Paläoanatomie München, Abt. Paläoanatomie, Staatliche Naturwissenschaftliche Sammlungen Bayerns (SAPM), Henriette Obermaier and Britta Möllenkamp.

Zoologisches Institut / Populationsgenetik (former Institut für Haustierkunde), Christian-Albrechts-Universität zu Kiel, Germany (I.f.H.), Renate Lücht.

France : Muséum national d’histoire Naturelle, Paris (MNHN), Aurélie Verguin, Celine Bens.

Austria: Naturhistorisches Museum, Wien (NHW), Frank Zachos and Alexander Bibl.

UK: University Museum Zoology Cambridge (UMZC), Robert Asher.

Russia: Zoological Institute of the Russian Academy of Sciences (ZIN), Gennady Baryshnikov, Alexander Averianov.

**Supplementary Information 1 B**. On the choice of species and populations sampled for the wild / domesticated comparisons

Here we provide further details on the sampling of taxa and populations in our study. We sympathize with the recommendations of Herre and Röhrs (1990) and Kruska and Steffen (2013) in having the same species name for the wild and the domestic form, with the latter being a forma ‘domestica’, but this use has not been universal and this is reflected in the names used.

It is widely accepted that the Przewalski’s horse is the best approximation to the wild form of the domestic horse, the European forest horse or Tarpan (Levine 2005; Jansen et al. 2002). All the horses sampled are Warmblood or Icelandic ones, as they represent the closest to a less-derived, first domesticated form (Hanot et al. 2017).

*Cavia tschudii* is the likely ancestral form of the guinea pig according to the most recent and comprehensive examination of this controversial matter (Dunnum and Salazar-Bravo 2010). *Cavia aperea*, which included in some older taxonomies what is now treated as *C. tschudii*, had been suggested as the wild form in older works (Müller-Haye 1984). As it is closely related to *Cavia tschudii* we use Museum specimens of wild *C. aperea* as an appropriate proxy for the former. Individuals of *C. tschudii* in collections to build a growth series are very few (we could collect data for only seven specimens for that purpose).

The wildcat has a broad geographic distribution and several subspecies have been recognized – recent work has hypothesized that the near Eastern wildcat *Felis silvestris lybica* is the ancestor of the domestic cat (Driscoll et al. 2007, 2009).

We used the Asian mouflon (*Ovis orientalis*; Hiendenlieber et al. 2002) as the wild form of the domestic sheep. This species is scarce in museum collections, so we added to our data from populations from Asia available in the collections in St. Petersburg (most individuals examined), a few individuals from introduced populations of this species from the island of Creta available in the visited collection in Munich. The European mouflon (*Ovis musimon*) is a Neolithic feral form of the first domestic sheep in Europe and thus not their wild ancestor (Meadows et al. 2011).

It is relevant to emphasize that hybridization has been a major factor in the evolution of domesticated forms at their origin and since then, and sorting out its effects is a major issue in studies of DNA (e.g., Larson and Fuller 2014), and has surely affected phenotypic patterns as well. Hybridization has been reportedly as important for many of the pairs examined in this work (e.g., South American camelids; Kadwell et al. 2001).

References for Supplementary Information 1B

Brooks SA, S. Makvandi-Nejad, E. Chu, J. J. Allen, C. Streeter, E. Gu, B. McCleery, B. A. Murphy, R. Bellone and N. B. Sutter. 2010. Morphological variation in the horse: defining complex traits of body size and shape. Animal Genetics, 41 (Suppl. 2), 159–165.

Hanot P, Guintard C, Lepetz S, Cornette R. 2017. Identifying domestic horses, donkeys and hybrids from archaeological deposits: A 3D morphological investigation on skeletons, Journal of Archaeological Science 78:88-98.

Meadows, J. R. S., S. Hiendleder, and J. W. Kijas. "Haplogroup relationships between domestic and wild sheep resolved using a mitogenome panel." Heredity 106.4 (2011): 700-706.

Müller-HayeB. 1984. Guinea pig or cuy. Pp. 252-257. In: Mason IL (ed) Evolution of domesticated mammals. 1984. Longmn House, Harlow.

**Supplementary information 1 C.** Range of size (geometric mean) and test of normality (Shapiro-Wilk test) in domestic and wild forms.

|  | Geometric mean range | *W* | *P* | *Degrees of Fredom* |  | |
| --- | --- | --- | --- | --- | --- | --- |
| *Canis lupus familiaris* | 1.94-8.45 | 0.92 | 0.33 | 100 |  |  |
| *Canis lupus lupus* | 3.95-8.92 | 0.84 | 0.18 | 23 |  |  |
| *Mustela putorius furo* | 1.28-2.18 | 0.92 | 0.35 | 58 |  |  |
| *Mustela putorius putorius* | 1.02-2.32 | 0.91 | 0.64 | 56 |  |  |
| *Neovison vison* | 1.63-2.22 | 0.95 | 0.65 | 13 |  |  |
| *Neovison vison letifera* | 1.69-2.39 | 0.97 | 0.61 | 60 |  |  |
| *Felis silvestris catus* | 1.29-3.79 | 0.86 | 0.79 | 133 |  |  |
| *Felis silvestris lybica* | 2.07-3.78 | 0.82 | 0.44 | 39 |  |  |
| *Equus ferus caballus* | 8.67-18.71 | 0.97 | 0.48 | 68 |  |  |
| *Equus ferus przewalskii* | 7.87-14.72 | 0.76 | 0.8 | 16 |  |  |
| *Capra hircus* | 3.02-7.61 | 0.89 | 0.27 | 48 |  |  |
| *Capra aegagrus* | 5.5-7.94 | 0.88 | 0.23 | 16 |  |  |
| *Ovis aries* | 3.74-8.75 | 0.94 | 0.64 | 42 |  |  |
| *Ovis musimon* | 3.04-7.77 | 0.95 | 0.78 | 30 |  |  |
| *Sus scrofa domestica* | 2.9-12.23 | 0.96 | 0.81 | 41 |  |  |
| *Sus scrofa scrofa* | 3.34-13.06 | 0.93 | 0.61 | 43 |  |  |
| *Lama glama* | 3.02-7.8 | 0.81 | 0.13 | 19 |  |  |
| *Lama guanicoe* | 4.36-8.4 | 0.82 | 0.4 | 26 |  |  |
| *Lama pacos* | 4-7.37 | 0.89 | 0.51 | 13 |  |  |
| *Vicugna vicugna* | 3.66-6.76 | 0.79 | 0.50 | 28 |  |  |
| *Camelus bactrianus* | 7.07-16.12 | 0.94 | 0.56 | 9 |  |  |
| *Camelus ferus* | 7.2-14.88 | 0.88 | 0.17 | 10 |  |  |
| *Oryctolagus cuniculus* f. *domesticus* | 1.19-3.5 | 0.89 | 0.71 | 23 |  |  |
| *Oryctolagus cuniculus* | 0.93-3.18 | 0.86 | 0.78 | 89 |  |  |
| *Cavia porcellus* | 1.37-2.26 | 0.98 | 0.89 | 42 |  |  |
| *Cavia aperea* | 1.24-2.21 | 0.98 | 0.83 | 46 |  |  |

**Supplementary information 1 D.** Description of measurements

Our measurements are mostly the same as those in recent studies of mammalian skull growth that are comprehensive in sampling and are currently being expanded (Flores et al. 2015), thus facilitating comparison of our results and future integration of datasets. In some cases small deviations had to be taken among species (but not within species, so the wild versus domestic forms could be compared with exactitude), but even in these cases the measurements were comparable in that they convey the same length or width of a portion of the skull, as it becomes evident in the descriptions below. Measurements are organized in the following sequence of regions: total skull length, splanchnocranial, neurocranial and lower jaw.

1. CPL: Condylo-premaxillary length. From the anterior tip of the premaxilla, to the posterior tip of the occipital condyle. As many taxa examined in this work lack incisors (goats, sheep, camels) we recorded this proxy for skull length independent of the presence of front teeth. In species without incisors and a gap between the two premaxillary bones (goats, sheep, camels), this measurement was approximated using the center of the virtual midline between the most distal parts of the premaxillary bones.

2. LN: Length of the nasal bones. From the anterior tip to the posterior point of the nasals, measured mesially on the internasalis suture.

3. HM: Height of the muzzle. From the anterior margin of the alveolous of the first postcanine (or first molariform) to the dorsal surface of the nasal bones. This measurement was also taken in very young specimens that had not yet any functional teeth (none of the teeth fully erupted). In these cases, the caliper was positioned at the anterior margin of the alveolus of the first not yet erupted postcanine. In *Camelus*we consistently measured the HM at the level of the first molariform – to avoid differences related to tooth eruption and to best reflect the height of the muzzle in this species.

4. UPR: Length of upper post-canine or of molariform toothrow. From the anterior margin of the alveolus of the first upper postcanine, to the posterior margin of the alveolus of the last upper tooth. In juvenile specimens, in which only the alveolus of the first post-canine tooth exists and the tooth element is in process of eruption, the alveolus is taken as an integral part of the toothrow and is included into this measurement. Neonates without any tooth elements in existence were excluded from the analysis of this variable.In *Camelus*the measurement UPR length of the upper-canine row refers to ‘molariforms’ only, excluding thus the simple tooth posterior to the canine, a premolar, that has not erupted in the smaller individuals examined.

5. LP: Length of palate. From posterior margin of the alveolus of the first incisive teeth (if present), to the middle point of the palatine torus (measured on interpalatine suture). In species without incisors and a gap between the two premaxillary bones (sheep, goat, camels), this measurement was approximated using the center of the virtual midline between the most distal parts of the premaxillary bones. In pigs, where the alveoli of the incisors is concealed by the teeth itself, this measurement was approximated as closely as possible.

6. BP: Breadth of palate. Maximum breadth of the palate measured between the internal margins of the upper tooth-row. This measurement is independent from the identity of the teeth between which this measurement is taken.

7. LO: Length of the orbit. From anterior point of the orbit to the infraorbitary process of the jugal or the posterior point of the postorbital bar.

8. ZB: Zygomatic breadth. Maximum breadth between the zygomatic arches.

9. BB: Breadth of the braincase. Maximum breadth of the braincase, measured on parietals, posterior to the base of the horns (if present). In *Camelus*, as the animals grow, the squamosal expands dorsally, in such a way that measuring the BB was done as the maximum width irrespective of the bone involved (in younger specimens the suture parietal-squamosal, in older ones the squamosal as in older ones it is in this bone where the maximum breadth is located).

10. HO: Height of occipital plate. From the superior margin of the occipital plate (including the sagittal crest, if present) to the base of the occipital condyle.

11. LD: Length of the dentary. A straight line from the most anterior point in the alveolus of the first functional incisor to the most posterior point of the dentary bone. This measurement is not homologous in specimens of different species, but in all cases serves to estimate the maximum length of the lower jaw, thus being functional homologous.

12. HD: Height of the dentary. The highest section of the horizontal ramus of the mandible. This measurement is not homologous in specimens of different age stages and species, but in all cases serves to estimate the maximum height of the horizontal portion of the dentary, thus being functionally similar.

13. HC: Height of the coronoid process. From the most superior part of the coronoid process to the base of the ascendant ramus of the mandible. This measurement is not homologous in specimens of different species, but in all cases serves to estimate the maximum height of the coronoid process, thus being functionally similar.

14. LPR: Length of lower post-canine toothrow. From the anterior margin of the alveolus of the first postcanine to the posterior margin of the alveolus of the last tooth. In juvenile specimens, in which only the alveolus of the first post-canine tooth exists and the tooth element is in process of eruption, the alveolus is taken as an integral part of the toothrow and is included into this measurement. Neonates without any tooth elements in existence were excluded from the analysis of this variable. In *Camelus* this measurement is of the molariform teeth only - analogous to what is described for UPR
