# Supplementary material for: On the lack of a universal pattern associated with mammalian domestication: differences in skull growth trajectories across phylogeny
Source: R Soc Open Sci. 2017 Oct 25;4(10):170876. doi: 10.1098/rsos.170876 (PMC5666271; doi:10.1098/rsos.170876)
Supplement: Supplementary Information 2 [file rsos170876supp2.docx]

**Supplementary Information 2.** Results of multivariate analyses of cranial allometry for the domesticated and wild forms investigated. The first three data columns show results using all specimens. The remaining columns show jackknife results calculated with untrimmed and trimmed (m = 1) sets of pseudovalues (see Giannini et al., 2004 for details). The allometric coefficient of a variable is the corresponding element of the first (unit) eigenvector per variable. The expected coefficient is the value under the assumption of isometry (0.267 for all variables, except for *Capra* and *Camelus*, where the expected coefficient values were 0.316 and 0.277 respectively). The observed coefficient is the value obtained with all specimens included. The resampled coefficient is the value generated by first-order jackknife resampling. Bias is the difference between the resampled and observed coefficients. The jackknifed 95% confidence interval (CI) is provided; allometric variables are those whose CIs exclude the expected value under the assumption of isometry (0.267). Abbreviations as in Fig. 2.ISO, isometry; POS, positive allometry, NEG, negative allometry.

|  |  |  |  | Untrimmed |  |  |  | Trimmed |  |  |  |
| --- | --- | --- | --- | --- | --- | --- | --- | --- | --- | --- | --- |
| Variable | Species | Observed | Departure | Resampled | Bias | 95% CI | Trend | Resampled | Bias | 95% CI | Trend |
| CPL | *Canis lupus familiaris* | 0.2798 | -0.0125 | 0.2813 | -0.0007 | 0.2742-0.2884 | POS | 0.2786 | 0.0006 | 0.2727-0.2844 | POS |
|  | *Canis lupus lupus* | 0.2705 | -0.0032 | 0.3021 | -0.0158 | 0.2573-0.3468 | ISO | 0.2617 | 0.0044 | 0.2582-0.2652 | NEG |
| LN | *Canis lupus familiaris* | 0.3620 | -0.0947 | 0.3635 | -0.0007 | 0.3490-0.3779 | POS | 0.3551 | 0.0034 | 0.3457-0.3645 | POS |
|  | *Canis lupus lupus* | 0.3664 | -0.0991 | 0.4137 | -0.0236 | 0.3434-0.4840 | POS | 0.3542 | 0.0061 | 0.3403-0.3681 | POS |
| HM | *Canis lupus familiaris* | 0.2224 | 0.0449 | 0.2242 | -0.0009 | 0.2123-0.2360 | NEG | 0.2203 | 0.0011 | 0.2103-0.2302 | NEG |
|  | *Canis lupus lupus* | 0.2513 | 0.0160 | 0.1354 | 0.0580 | -0.0658-0.3367 | ISO | 0.3134 | -0.0310 | 0.2927-0.3341 | POS |
| URP | *Canis lupus familiaris* | 0.3529 | -0.0856 | 0.3498 | 0.0015 | 0.3295-0.3702 | POS | 0.3585 | -0.0028 | 0.3415-0.3755 | POS |
|  | *Canis lupus lupus* | 0.3161 | -0.0488 | 0.4648 | -0.0744 | 0.2253-0.7044 | ISO | 0.2478 | 0.0341 | 0.2282-0.2675 | ISO |
| LP | *Canis lupus familiaris* | 0.2688 | -0.0015 | 0.2700 | -0.0006 | 0.2632-0.2767 | ISO | 0.2681 | 0.0004 | 0.2624-0.2738 | ISO |
|  | *Canis lupus lupus* | 0.2517 | 0.0156 | 0.2558 | -0.0021 | 0.2468-0.2648 | NEG | 0.2552 | -0.0018 | 0.2489-0.2616 | NEG |
| BP | *Canis lupus familiaris* | 0.1859 | 0.0814 | 0.1892 | -0.0016 | 0.1680-0.2105 | NEG | 0.1841 | 0.0009 | 0.1662-0.2019 | NEG |
|  | *Canis lupus lupus* | 0.2007 | 0.0666 | 0.2234 | -0.0113 | 0.1793-0.2674 | ISO | 0.2097 | -0.0045 | 0.1932-0.2263 | NEG |
| LO | *Canis lupus familiaris* | 0.1913 | 0.0760 | 0.1929 | -0.0008 | 0.1760-0.2099 | NEG | 0.1944 | -0.0015 | 0.1848-0.2040 | NEG |
|  | *Canis lupus lupus* | 0.1908 | 0.0765 | 0.1704 | 0.0102 | 0.1279-0.2130 | NEG | 0.2020 | -0.0056 | 0.1902-0.2138 | NEG |
| ZB | *Canis lupus familiaris* | 0.2157 | 0.0516 | 0.2174 | -0.0008 | 0.2064-0.2283 | NEG | 0.2144 | 0.0007 | 0.2053-0.2234 | NEG |
|  | *Canis lupus lupus* | 0.2863 | -0.0190 | 0.1974 | 0.0445 | 0.0382-0.3566 | ISO | 0.3442 | -0.0289 | 0.3289-0.3595 | POS |
| BB | *Canis lupus familiaris* | 0.0953 | 0.1719 | 0.1000 | -0.0024 | 0.0711-0.1290 | NEG | 0.0928 | 0.0013 | 0.0680-0.1177 | NEG |
|  | *Canis lupus lupus* | 0.0015 | 0.2658 | -0.1949 | 0.0982 | -0.3287-(-0.0610) | NEG | -0.0723 | 0.0369 | -0.0946-(-0.0500) | NEG |
| HO | *Canis lupus familiaris* | 0.2218 | 0.0455 | 0.2263 | -0.0022 | 0.2006-0.2519 | NEG | 0.2120 | 0.0049 | 0.1924-0.2316 | NEG |
|  | *Canis lupus lupus* | 0.2972 | -0.0299 | 0.2991 | -0.0010 | 0.2758-0.3224 | POS | 0.3079 | -0.0054 | 0.2915-0.3243 | POS |
| LD | *Canis lupus familiaris* | 0.2827 | -0.0154 | 0.2838 | -0.0005 | 0.2789-0.2887 | POS | 0.2827 | 0.0000 | 0.2783-0.2871 | POS |
|  | *Canis lupus lupus* | 0.2846 | -0.0173 | 0.3131 | -0.0142 | 0.2734-0.3528 | POS | 0.2787 | 0.0029 | 0.2716-0.2858 | POS |
| HD | *Canis lupus familiaris* | 0.2049 | 0.0624 | 0.2083 | -0.0017 | 0.1901-0.2265 | NEG | 0.2021 | 0.0014 | 0.1870-0.2172 | NEG |
|  | *Canis lupus lupus* | 0.2613 | 0.0060 | 0.2403 | 0.0105 | 0.1900-0.2906 | ISO | 0.2613 | 0.0000 | 0.2391-0.2836 | ISO |
| HC | *Canis lupus familiaris* | 0.3234 | -0.0561 | 0.3229 | 0.0002 | 0.3140-0.3318 | POS | 0.3257 | -0.0012 | 0.3185-0.3330 | POS |
|  | *Canis lupus lupus* | 0.3489 | -0.0816 | 0.3474 | 0.0007 | 0.3299-0.3649 | POS | 0.3530 | -0.0021 | 0.3435-0.3626 | POS |
| LPR | *Canis lupus familiaris* | 0.3767 | -0.1094 | 0.3693 | 0.0037 | 0.3283-0.4104 | POS | 0.3835 | -0.0034 | 0.3471-0.4200 | POS |
|  | *Canis lupus lupus* | 0.2195 | 0.0478 | 0.3142 | -0.0474 | 0.1613-0.4672 | ISO | 0.1776 | 0.0209 | 0.1598-0.1954 | NEG |

|  |  | |  | |  | | Untrimmed | | |  |  | | |  | Trimmed | | |  |  | | |  |
| --- | --- | --- | --- | --- | --- | --- | --- | --- | --- | --- | --- | --- | --- | --- | --- | --- | --- | --- | --- | --- | --- | --- |
| Variable | Species | | Observed | | Departure | | Resampled | | | Bias | 95% CI | | | Trend | Resampled | | | Bias | 95% CI | | | Trend |
| CPL | *Caprahircus* | | 0.3418 | | -0.0256 | | 0.3420 | | | -0.0001 | 0.3316-0.3523 | | | POS | 0.3408 | | | 0.0005 | 0.3321-0.3496 | | | POS |
|  | *Capraaegagrus* | | 0.3048 | | 0.0114 | | 0.3111 | | | -0.0032 | 0.2292-0.3930 | | | ISO | 0.2722 | | | 0.0163 | 0.2212-0.3232 | | | ISO |
| LN | *Caprahircus* | | 0.4910 | | -0.1748 | | 0.4919 | | | -0.0004 | 0.4607-0.5230 | | | POS | 0.4910 | | | 0.0000 | 0.4638-0.5183 | | | POS |
|  | *Capraaegagrus* | | 0.2450 | | 0.0712 | | 0.2103 | | | 0.0174 | -0.0978-0.5183 | | | ISO | 0.0775 | | | 0.0838 | -0.0496-0.2046 | | | NEG |
| HM | *Caprahircus* | | 0.3729 | | -0.0567 | | 0.3732 | | | -0.0001 | 0.3462-0.4003 | | | POS | 0.3803 | | | -0.0037 | 0.3583-0.4024 | | | POS |
|  | *Capraaegagrus* | | 0.4245 | | -0.1083 | | 0.4578 | | | -0.0166 | 0.3012-0.6143 | | | ISO | 0.3528 | | | 0.0359 | 0.2907-0.4149 | | | ISO |
| URP | *Caprahircus* | | 0.3199 | | -0.0037 | | 0.3176 | | | 0.0011 | 0.2873-0.3479 | | | ISO | 0.3243 | | | -0.0022 | 0.2977-0.3508 | | | ISO |
|  | *Capraaegagrus* | | 0.3129 | | 0.0033 | | 0.3212 | | | -0.0042 | 0.1917-0.4508 | | | ISO | 0.3177 | | | -0.0024 | 0.2718-0.3636 | | | ISO |
| LP | *Caprahircus* | | 0.3763 | | -0.0601 | | 0.3770 | | | -0.0003 | 0.3614-0.3925 | | | POS | 0.3695 | | | 0.0034 | 0.3595-0.3795 | | | POS |
|  | *Capraaegagrus* | | 0.3138 | | 0.0024 | | 0.3081 | | | 0.0028 | 0.1606-0.4556 | | | ISO | 0.2384 | | | 0.0377 | 0.1559-0.3209 | | | ISO |
| BP | *Caprahircus* | | 0.2535 | | 0.0627 | | 0.2555 | | | -0.0010 | 0.2234-0.2876 | | | NEG | 0.2510 | | | 0.0013 | 0.2250-0.2769 | | | NEG |
|  | *Capraaegagrus* | | 0.2968 | | 0.0194 | | 0.3082 | | | -0.0057 | 0.2170-0.3994 | | | ISO | 0.2872 | | | 0.0048 | 0.2241-0.3504 | | | ISO |
| LO | *Caprahircus* | | 0.1798 | | 0.1364 | | 0.1811 | | | -0.0006 | 0.1594-0.2027 | | | NEG | 0.1782 | | | 0.0008 | 0.1625-0.1938 | | | NEG |
| *Capraaegagrus* | | 0.1530 | | 0.1632 | | 0.1617 | | -0.0044 | 0.1322-0.1913 | | | NEG | 0.1641 | | | -0.0056 | 0.1404-0.1878 | | | NEG |  |  |
| ZB | *Caprahircus* | | 0.2678 | | 0.0484 | | 0.2688 | | | -0.0005 | 0.2491-0.2884 | | | NEG | 0.2743 | | | -0.0032 | 0.2577-0.2909 | | | NEG |
|  | *Capraaegagrus* | | 0.2443 | | 0.0719 | | 0.2450 | | | -0.0004 | 0.1438-0.3463 | | | ISO | 0.1912 | | | 0.0266 | 0.1249-0.2575 | | | NEG |
| BB | *Caprahircus* | | 0.1359 | | 0.1803 | | 0.1359 | | | 0.0000 | 0.1108-0.1611 | | | NEG | 0.1361 | | | -0.0001 | 0.1136-0.1587 | | | NEG |
|  | *Capraaegagrus* | | 0.1723 | | 0.1439 | | 0.1876 | | | -0.0076 | 0.1100-0.2651 | | | NEG | 0.1796 | | | -0.0036 | 0.1196-0.2395 | | | NEG |
| HO | *Caprahircus* | | 0.2689 | | 0.0473 | | 0.2708 | | | -0.0009 | 0.2372-0.3044 | | | NEG | 0.2623 | | | 0.0033 | 0.2372-0.2875 | | | NEG |
|  | *Capraaegagrus* | | 0.5192 | | -0.2030 | | 0.6188 | | | -0.0498 | 0.2727-0.9650 | | | ISO | 0.7862 | | | -0.1335 | 0.5822-0.9901 | | | POS |

|  |  |  |  | Untrimmed |  |  |  | Trimmed |  |  |  |
| --- | --- | --- | --- | --- | --- | --- | --- | --- | --- | --- | --- |
| Variable | Species | Observed | Departure | Resampled | Bias | 95% CI | Trend | Resampled | Bias | 95% CI | Trend |
| CPL | *Cavia porcellus* | 0.2626 | 0.0047 | 0.2610 | 0.0008 | 0.2207-0.3013 | ISO | 0.2727 | -0.0050 | 0.2420-0.3034 | ISO |
|  | *Cavia aperea* | 0.2699 | -0.0026 | 0.2705 | -0.0003 | 0.2520-0.2890 | ISO | 0.2739 | -0.0020 | 0.2622-0.2856 | ISO |
| LN | *Cavia porcellus* | 0.2637 | 0.0036 | 0.2572 | 0.0032 | 0.1776-0.3368 | ISO | 0.2949 | -0.0156 | 0.2534-0.3363 | ISO |
|  | *Cavia aperea* | 0.4112 | -0.1439 | 0.4246 | -0.0067 | 0.3429-0.5063 | POS | 0.3641 | 0.0235 | 0.3299-0.3983 | POS |
| HM | *Cavia porcellus* | 0.2596 | 0.0077 | 0.2602 | -0.0003 | 0.2344-0.2860 | ISO | 0.2620 | -0.0012 | 0.2430-0.2811 | ISO |
|  | *Cavia aperea* | 0.2666 | 0.0007 | 0.2648 | 0.0009 | 0.2369-0.2927 | ISO | 0.2758 | -0.0046 | 0.2573-0.2944 | ISO |
| URP | *Cavia porcellus* | 0.2294 | 0.0379 | 0.2325 | -0.0015 | 0.2060-0.2589 | NEG | 0.2271 | 0.0011 | 0.2071-0.2471 | NEG |
|  | *Cavia aperea* | 0.2463 | 0.0210 | 0.2480 | -0.0009 | 0.2281-0.2679 | ISO | 0.2505 | -0.0021 | 0.2342-0.2668 | NEG |
| LP | *Cavia porcellus* | 0.2981 | -0.0308 | 0.2936 | 0.0022 | 0.2313-0.3559 | ISO | 0.3369 | -0.0194 | 0.3132-0.3605 | POS |
|  | *Cavia aperea* | 0.3231 | -0.0558 | 0.3254 | -0.0011 | 0.3076-0.3431 | POS | 0.3256 | -0.0012 | 0.3110-0.3402 | POS |
| BP | *Cavia porcellus* | 0.5003 | -0.2330 | 0.5144 | -0.0071 | 0.4001-0.6287 | POS | 0.4723 | 0.0140 | 0.4189-0.5257 | POS |
|  | *Cavia aperea* | 0.3474 | -0.0801 | 0.3468 | 0.0003 | 0.2654-0.4282 | ISO | 0.3386 | 0.0044 | 0.2697-0.4075 | POS |
| LO | *Cavia porcellus* | 0.1796 | 0.0877 | 0.1848 | -0.0026 | 0.1367-0.2330 | NEG | 0.1544 | 0.0126 | 0.1299-0.1790 | NEG |
|  | *Cavia aperea* | 0.2107 | 0.0566 | 0.2127 | -0.0010 | 0.1819-0.2435 | NEG | 0.2120 | -0.0007 | 0.1872-0.2369 | NEG |
| ZB | *Cavia porcellus* | 0.2409 | 0.0264 | 0.2386 | 0.0011 | 0.1894-0.2878 | ISO | 0.2603 | -0.0097 | 0.2380-0.2825 | ISO |
|  | *Cavia aperea* | 0.2837 | -0.0164 | 0.2856 | -0.0009 | 0.2668-0.3044 | ISO | 0.2802 | 0.0018 | 0.2653-0.2952 | ISO |
| BB | *Cavia porcellus* | 0.0516 | 0.2157 | 0.0562 | -0.0023 | 0.0224-0.0901 | NEG | 0.0556 | -0.0020 | 0.0274-0.0837 | NEG |
|  | *Cavia aperea* | 0.1312 | 0.1361 | 0.1288 | 0.0012 | 0.1050-0.1526 | NEG | 0.1403 | -0.0045 | 0.1231-0.1575 | NEG |
| HO | *Cavia porcellus* | 0.1481 | 0.1192 | 0.1535 | -0.0027 | 0.1169-0.1902 | NEG | 0.1405 | 0.0038 | 0.1145-0.1665 | NEG |
|  | *Cavia aperea* | 0.1833 | 0.0840 | 0.1803 | 0.0015 | 0.1464-0.2143 | NEG | 0.1906 | -0.0036 | 0.1653-0.2158 | NEG |
| LD | *Cavia porcellus* | 0.2355 | 0.0318 | 0.2389 | -0.0017 | 0.2102-0.2675 | ISO | 0.2346 | 0.0004 | 0.2130-0.2563 | NEG |
|  | *Cavia aperea* | 0.2848 | -0.0175 | 0.2863 | -0.0008 | 0.2599-0.3128 | ISO | 0.2874 | -0.0013 | 0.2660-0.3089 | ISO |
| HD | *Cavia porcellus* | 0.3030 | -0.0357 | 0.3112 | -0.0041 | 0.2486-0.3738 | ISO | 0.3013 | 0.0008 | 0.2505-0.3521 | ISO |
|  | *Cavia aperea* | 0.1861 | 0.0812 | 0.1842 | 0.0009 | 0.1143-0.2541 | NEG | 0.1749 | 0.0056 | 0.1125-0.2374 | NEG |
| HC | *Cavia porcellus* | 0.2933 | -0.0260 | 0.2925 | 0.0004 | 0.2522-0.3328 | ISO | 0.2972 | -0.0020 | 0.2628-0.3316 | ISO |
|  | *Cavia aperea* | 0.2561 | 0.0112 | 0.2596 | -0.0017 | 0.2202-0.2990 | ISO | 0.2544 | 0.0008 | 0.2261-0.2827 | ISO |
| LPR | *Cavia porcellus* | 0.2330 | 0.0343 | 0.2375 | -0.0023 | 0.2062-0.2688 | ISO | 0.2335 | -0.0003 | 0.2091-0.2579 | NEG |
|  | *Cavia aperea* | 0.2104 | 0.0569 | 0.2073 | 0.0015 | 0.1726-0.2420 | NEG | 0.2227 | -0.0062 | 0.2036-0.2417 | NEG |

|  |  |  |  | Untrimmed |  |  |  | Trimmed |  |  |  |
| --- | --- | --- | --- | --- | --- | --- | --- | --- | --- | --- | --- |
| Variable | Species | Observed | Departure | Resampled | Bias | 95% CI | Trend | Resampled | Bias | 95% CI | Trend |
| CPL | *Equusferuscaballus* | 0.2852 | -0.0179 | 0.2861 | -0.0005 | 0.2738-0.2984 | POS | 0.2897 | -0.0023 | 0.2812-0.2982 | POS |
|  | *Equusferusprzewalskii* | 0.2602 | 0.0071 | 0.2607 | -0.0002 | 0.2498-0.2716 | ISO | 0.2605 | -0.0002 | 0.2522-0.2689 | ISO |
| LN | *Equusferuscaballus* | 0.3358 | -0.0685 | 0.3332 | 0.0013 | 0.2966-0.3697 | POS | 0.3498 | -0.0070 | 0.3251-0.3745 | POS |
|  | *Equusferusprzewalskii* | 0.3441 | -0.0768 | 0.3416 | 0.0012 | 0.2950-0.3882 | POS | 0.3652 | -0.0105 | 0.3401-0.3903 | POS |
| HM | *Equusferuscaballus* | 0.3565 | -0.0892 | 0.3532 | 0.0016 | 0.3153-0.3911 | POS | 0.3773 | -0.0104 | 0.3598-0.3947 | POS |
|  | *Equusferusprzewalskii* | 0.3425 | -0.0752 | 0.3431 | -0.0003 | 0.3136-0.3726 | POS | 0.3505 | -0.0040 | 0.3313-0.3698 | POS |
| URP | *Equusferuscaballus* | 0.2614 | 0.0059 | 0.2614 | 0.0000 | 0.2158-0.3069 | ISO | 0.2522 | 0.0046 | 0.2148-0.2897 | ISO |
|  | *Equusferusprzewalskii* | 0.2442 | 0.0231 | 0.2418 | 0.0012 | 0.2126-0.2710 | ISO | 0.2499 | -0.0029 | 0.2266-0.2732 | ISO |
| LP | *Equusferuscaballus* | 0.3144 | -0.0471 | 0.3149 | -0.0002 | 0.2996-0.3301 | POS | 0.3212 | -0.0034 | 0.3108-0.3317 | POS |
|  | *Equusferusprzewalskii* | 0.3026 | -0.0353 | 0.3055 | -0.0015 | 0.2845-0.3266 | POS | 0.2940 | 0.0043 | 0.2806-0.3075 | POS |
| BP | *Equusferuscaballus* | 0.2192 | 0.0481 | 0.2206 | -0.0007 | 0.2006-0.2407 | NEG | 0.2194 | -0.0001 | 0.2022-0.2366 | NEG |
|  | *Equusferusprzewalskii* | 0.2516 | 0.0157 | 0.2532 | -0.0008 | 0.2303-0.2760 | ISO | 0.2541 | -0.0013 | 0.2340-0.2742 | ISO |
| LO | *Equusferuscaballus* | 0.1691 | 0.0982 | 0.1767 | -0.0038 | 0.1246-0.2288 | NEG | 0.1549 | 0.0071 | 0.1276-0.1822 | NEG |
|  | *Equusferusprzewalskii* | 0.1371 | 0.1302 | 0.1408 | -0.0019 | 0.0981-0.1836 | NEG | 0.1255 | 0.0058 | 0.1032-0.1478 | NEG |
| ZB | *Equusferuscaballus* | 0.2100 | 0.0573 | 0.2136 | -0.0018 | 0.1943-0.2329 | NEG | 0.2042 | 0.0029 | 0.1941-0.2144 | NEG |
|  | *Equusferusprzewalskii* | 0.2547 | 0.0126 | 0.2566 | -0.0010 | 0.2264-0.2869 | ISO | 0.2533 | 0.0007 | 0.2407-0.2660 | NEG |
| BB | *Equusferuscaballus* | 0.0643 | 0.2030 | 0.0677 | -0.0017 | 0.0370-0.0985 | NEG | 0.0569 | 0.0037 | 0.0329-0.0808 | NEG |
|  | *Equusferusprzewalskii* | 0.0621 | 0.2051 | 0.0625 | -0.0002 | 0.0420-0.0831 | NEG | 0.0637 | -0.0008 | 0.0468-0.0805 | NEG |
| HO | *Equusferuscaballus* | 0.2459 | 0.0214 | 0.2494 | -0.0018 | 0.2144-0.2845 | ISO | 0.2446 | 0.0006 | 0.2194-0.2699 | ISO |
|  | *Equusferusprzewalskii* | 0.1754 | 0.0919 | 0.1814 | -0.0030 | 0.1254-0.2375 | NEG | 0.1600 | 0.0077 | 0.1220-0.1980 | NEG |
| LD | *Equusferuscaballus* | 0.2797 | -0.0124 | 0.2799 | -0.0001 | 0.2673-0.2924 | POS | 0.2813 | -0.0008 | 0.2714-0.2912 | POS |
|  | *Equusferusprzewalskii* | 0.2707 | -0.0034 | 0.2709 | -0.0001 | 0.2586-0.2832 | ISO | 0.2726 | -0.0010 | 0.2622-0.2831 | ISO |
| HD | *Equusferuscaballus* | 0.2421 | 0.0252 | 0.2503 | -0.0041 | 0.2014-0.2992 | ISO | 0.2324 | 0.0048 | 0.2072-0.2577 | NEG |
|  | *Equusferusprzewalskii* | 0.2844 | -0.0171 | 0.2873 | -0.0015 | 0.2587-0.3160 | ISO | 0.2789 | 0.0028 | 0.2588-0.2989 | ISO |
| HC | *Equusferuscaballus* | 0.3440 | -0.0767 | 0.3435 | 0.0002 | 0.3180-0.3690 | POS | 0.3552 | -0.0056 | 0.3365-0.3738 | POS |
|  | *Equusferusprzewalskii* | 0.3834 | -0.1161 | 0.3851 | -0.0008 | 0.3633-0.4069 | POS | 0.3839 | -0.0003 | 0.3676-0.4003 | POS |
| LPR | *Equusferuscaballus* | 0.2669 | 0.0004 | 0.2681 | -0.0006 | 0.2230-0.3132 | ISO | 0.2560 | 0.0055 | 0.2193-0.2927 | ISO |
|  | *Equusferusprzewalskii* | 0.2503 | 0.0170 | 0.2473 | 0.0015 | 0.2140-0.2806 | ISO | 0.2579 | -0.0038 | 0.2329-0.2829 | ISO |

|  |  |  |  | Untrimmed |  |  |  | Trimmed |  |  |  |
| --- | --- | --- | --- | --- | --- | --- | --- | --- | --- | --- | --- |
| Variable | Species | Observed | Departure | Resampled | Bias | 95% CI | Trend | Resampled | Bias | 95% CI | Trend |
| CPL | *Felissilvestriscatus* | 0.2694 | -0.0021 | 0.2696 | -0.0001 | 0.2648-0.2743 | ISO | 0.2697 | -0.0002 | 0.2653-0.2741 | ISO |
|  | *Felissilvestrislybica* | 0.2441 | 0.0232 | 0.2440 | 0.0001 | 0.2270-0.2610 | NEG | 0.2448 | -0.0003 | 0.2317-0.2579 | NEG |
| LN | *Felissilvestriscatus* | 0.2948 | -0.0275 | 0.2957 | -0.0005 | 0.2804-0.3111 | POS | 0.2917 | 0.0015 | 0.2789-0.3046 | POS |
|  | *Felissilvestrislybica* | 0.2978 | -0.0305 | 0.3376 | -0.0199 | 0.2369-0.4382 | ISO | 0.2768 | 0.0105 | 0.2432-0.3103 | ISO |
| HM | *Felissilvestriscatus* | 0.2451 | 0.0222 | 0.2451 | 0.0000 | 0.2164-0.2737 | ISO | 0.2463 | -0.0006 | 0.2211-0.2715 | ISO |
|  | *Felissilvestrislybica* | 0.2783 | -0.0110 | 0.3291 | -0.0254 | 0.1927-0.4654 | ISO | 0.2775 | 0.0004 | 0.1875-0.3676 | ISO |
| URP | *Felissilvestriscatus* | 0.2461 | 0.0212 | 0.2456 | 0.0003 | 0.2285-0.2627 | NEG | 0.2476 | -0.0007 | 0.2332-0.2620 | NEG |
|  | *Felissilvestrislybica* | 0.2489 | 0.0184 | 0.2850 | -0.0180 | 0.1926-0.3773 | ISO | 0.2404 | 0.0043 | 0.1726-0.3082 | ISO |
| LP | *Felissilvestriscatus* | 0.2707 | -0.0034 | 0.2706 | 0.0001 | 0.2644-0.2769 | ISO | 0.2722 | -0.0007 | 0.2668-0.2775 | ISO |
|  | *Felissilvestrislybica* | 0.2635 | 0.0038 | 0.2811 | -0.0088 | 0.2258-0.3364 | ISO | 0.2661 | -0.0013 | 0.2350-0.2973 | ISO |
| BP | *Felissilvestriscatus* | 0.2959 | -0.0286 | 0.2975 | -0.0008 | 0.2721-0.3229 | POS | 0.2968 | -0.0005 | 0.2761-0.3176 | POS |
|  | *Felissilvestrislybica* | 0.2698 | -0.0025 | 0.3015 | -0.0158 | 0.2335-0.3695 | ISO | 0.2502 | 0.0098 | 0.2242-0.2763 | ISO |
| LO | *Felissilvestriscatus* | 0.2703 | -0.0030 | 0.2707 | -0.0002 | 0.2578-0.2836 | ISO | 0.2646 | 0.0028 | 0.2572-0.2720 | ISO |
|  | *Felissilvestrislybica* | 0.2251 | 0.0422 | 0.2330 | -0.0040 | 0.1927-0.2733 | ISO | 0.2194 | 0.0029 | 0.1947-0.2440 | NEG |
| ZB | *Felissilvestriscatus* | 0.2852 | -0.0179 | 0.2857 | -0.0003 | 0.2797-0.2918 | POS | 0.2848 | 0.0002 | 0.2795-0.2902 | POS |
|  | *Felissilvestrislybica* | 0.2696 | -0.0023 | 0.2652 | 0.0022 | 0.2391-0.2913 | ISO | 0.2754 | -0.0029 | 0.2586-0.2923 | ISO |
| BB | *Felissilvestriscatus* | 0.1015 | 0.1658 | 0.1038 | -0.0012 | 0.0816-0.1261 | NEG | 0.0985 | 0.0015 | 0.0789-0.1181 | NEG |
|  | *Felissilvestrislybica* | 0.0740 | 0.1932 | 0.0789 | -0.0024 | 0.0637-0.0940 | NEG | 0.0779 | -0.0019 | 0.0654-0.0904 | NEG |
| HO | *Felissilvestriscatus* | 0.1959 | 0.0714 | 0.1969 | -0.0005 | 0.1834-0.2105 | NEG | 0.1921 | 0.0019 | 0.1817-0.2025 | NEG |
|  | *Felissilvestrislybica* | 0.1763 | 0.0910 | 0.1514 | 0.0125 | 0.0779-0.2249 | NEG | 0.2091 | -0.0164 | 0.1854-0.2328 | NEG |
| LD | *Felissilvestriscatus* | 0.2947 | -0.0274 | 0.2946 | 0.0001 | 0.2870-0.3022 | POS | 0.2975 | -0.0014 | 0.2922-0.3028 | POS |
|  | *Felissilvestrislybica* | 0.2885 | -0.0212 | 0.2831 | 0.0027 | 0.2536-0.3125 | ISO | 0.2940 | -0.0028 | 0.2780-0.3100 | POS |
| HD | *Felissilvestriscatus* | 0.2959 | -0.0286 | 0.2952 | 0.0004 | 0.2808-0.3095 | POS | 0.2964 | -0.0002 | 0.2840-0.3087 | POS |
|  | *Felissilvestrislybica* | 0.3336 | -0.0663 | 0.2749 | 0.0294 | 0.1172-0.4326 | ISO | 0.3964 | -0.0314 | 0.3511-0.4417 | POS |
| HC | *Felissilvestriscatus* | 0.3611 | -0.0938 | 0.3604 | 0.0003 | 0.3467-0.3740 | POS | 0.3637 | -0.0013 | 0.3523-0.3751 | POS |
|  | *Felissilvestrislybica* | 0.3894 | -0.1221 | 0.3584 | 0.0155 | 0.2683-0.4485 | POS | 0.4341 | -0.0223 | 0.4085-0.4596 | POS |
| LPR | *Felissilvestriscatus* | 0.2264 | 0.0409 | 0.2269 | -0.0002 | 0.2072-0.2465 | NEG | 0.2199 | 0.0032 | 0.2059-0.2339 | NEG |
|  | *Felissilvestrislybica* | 0.2530 | 0.0143 | 0.3068 | -0.0269 | 0.1840-0.4296 | ISO | 0.2020 | 0.0255 | 0.1798-0.2242 | NEG |

|  |  |  |  | Untrimmed |  |  |  | Trimmed |  |  |  |
| --- | --- | --- | --- | --- | --- | --- | --- | --- | --- | --- | --- |
| Variable | Species | Observed | Departure | Resampled | Bias | 95% CI | Trend | Resampled | Bias | 95% CI | Trend |
| CPL | *Lama pacos* | 0.2127 | 0.0546 | 0.2311 | -0.0092 | 0.1941-0.2681 | ISO | 0.2140 | -0.0007 | 0.1958-0.2323 | NEG |
|  | *Vicugnavicugna* | 0.2657 | 0.0016 | 0.2792 | -0.0068 | 0.2433-0.3152 | ISO | 0.2603 | 0.0027 | 0.2504-0.2701 | ISO |
| LN | *Lama pacos* | 0.7476 | -0.4803 | 0.7098 | 0.0189 | 0.5143-0.9052 | POS | 0.8007 | -0.0266 | 0.6967-0.9048 | POS |
|  | *Vicugnavicugna* | 0.5524 | -0.2851 | 0.5528 | -0.0002 | 0.4204-0.6852 | POS | 0.5687 | -0.0082 | 0.5136-0.6238 | POS |
| HM | *Lama pacos* | 0.1945 | 0.0728 | 0.2856 | -0.0455 | 0.0815-0.4898 | ISO | 0.1834 | 0.0056 | 0.0972-0.2697 | ISO |
|  | *Vicugnavicugna* | 0.2380 | 0.0293 | 0.2508 | -0.0064 | 0.1847-0.3169 | ISO | 0.2384 | -0.0002 | 0.2039-0.2730 | ISO |
| URP | *Lama pacos* | 0.2476 | 0.0197 | 0.3502 | -0.0513 | 0.0960-0.6044 | ISO | 0.1537 | 0.0470 | 0.1186-0.1888 | NEG |
|  | *Vicugnavicugna* | 0.2757 | -0.0084 | 0.2696 | 0.0031 | 0.2213-0.3179 | ISO | 0.2691 | 0.0033 | 0.2371-0.3012 | ISO |
| LP | *Lama pacos* | 0.2763 | -0.0090 | 0.3035 | -0.0136 | 0.2461-0.3609 | ISO | 0.2668 | 0.0047 | 0.2431-0.2905 | ISO |
|  | *Vicugnavicugna* | 0.3882 | -0.1209 | 0.4099 | -0.0108 | 0.2322-0.5876 | ISO | 0.3442 | 0.0220 | 0.3228-0.3657 | POS |
| BP | *Lama pacos* | 0.1268 | 0.1405 | 0.1499 | -0.0116 | 0.0591-0.2407 | NEG | 0.1308 | -0.0020 | 0.0752-0.1863 | NEG |
|  | *Vicugnavicugna* | 0.0617 | 0.2055 | 0.0477 | 0.0070 | -0.0179-0.1133 | NEG | 0.0797 | -0.0090 | 0.0459-0.1135 | NEG |
| LO | *Lama pacos* | 0.1799 | 0.0874 | 0.2092 | -0.0146 | 0.1130-0.3054 | ISO | 0.1569 | 0.0115 | 0.1179-0.1960 | NEG |
|  | *Vicugnavicugna* | 0.1163 | 0.1510 | 0.1183 | -0.0010 | 0.0920-0.1447 | NEG | 0.1158 | 0.0002 | 0.0973-0.1343 | NEG |
| ZB | *Lama pacos* | 0.1708 | 0.0965 | 0.2005 | -0.0149 | 0.1451-0.2559 | NEG | 0.1705 | 0.0002 | 0.1333-0.2077 | NEG |
|  | *Vicugnavicugna* | 0.1796 | 0.0877 | 0.1996 | -0.0100 | 0.1265-0.2726 | ISO | 0.1612 | 0.0092 | 0.1469-0.1755 | NEG |
| BB | *Lama pacos* | 0.0845 | 0.1828 | 0.0573 | 0.0136 | -0.0339-0.1485 | NEG | 0.0842 | 0.0001 | 0.0296-0.1389 | NEG |
|  | *Vicugnavicugna* | 0.0195 | 0.2478 | 0.0253 | -0.0029 | -0.0003-0.0508 | NEG | 0.0116 | 0.0039 | -0.0041-0.0274 | NEG |
| HO | *Lama pacos* | 0.0953 | 0.1720 | 0.0870 | 0.0041 | 0.0188-0.1552 | NEG | 0.1127 | -0.0087 | 0.0796-0.1458 | NEG |
|  | *Vicugnavicugna* | 0.1454 | 0.1219 | 0.1783 | -0.0164 | 0.0470-0.3096 | ISO | 0.1054 | 0.0200 | 0.0837-0.1270 | NEG |
| LD | *Lama pacos* | 0.1897 | 0.0776 | 0.1987 | -0.0045 | 0.1733-0.2242 | NEG | 0.1954 | -0.0029 | 0.1755-0.2154 | NEG |
|  | *Vicugnavicugna* | 0.2662 | 0.0011 | 0.2814 | -0.0076 | 0.2349-0.3279 | ISO | 0.2592 | 0.0035 | 0.2433-0.2751 | ISO |
| HD | *Lama pacos* | 0.1576 | 0.1097 | 0.2856 | -0.0640 | -0.0583-0.6294 | ISO | 0.0819 | 0.0379 | -0.0418-0.2055 | NEG |
|  | *Vicugnavicugna* | 0.1545 | 0.1128 | 0.1200 | 0.0173 | 0.0025-0.2374 | NEG | 0.1856 | -0.0155 | 0.1440-0.2271 | NEG |
| HC | *Lama pacos* | 0.1617 | 0.1056 | 0.2040 | -0.0211 | 0.1196-0.2883 | ISO | 0.1548 | 0.0034 | 0.1255-0.1842 | NEG |
|  | *Vicugnavicugna* | 0.3119 | -0.0446 | 0.3385 | -0.0133 | 0.2668-0.4101 | ISO | 0.2887 | 0.0116 | 0.2643-0.3131 | ISO |
| LPR | *Lama pacos* | 0.1990 | 0.0683 | 0.2096 | -0.0053 | 0.1706-0.2485 | NEG | 0.2079 | -0.0044 | 0.1829-0.2329 | NEG |
|  | *Vicugnavicugna* | 0.2790 | -0.0117 | 0.2875 | -0.0043 | 0.1365-0.4385 | ISO | 0.3021 | -0.0116 | 0.2569-0.3473 | ISO |

|  |  |  |  | Untrimmed |  |  |  | Trimmed |  |  |  |
| --- | --- | --- | --- | --- | --- | --- | --- | --- | --- | --- | --- |
| Variable | Species | Observed | Departure | Resampled | Bias | 95% CI | Trend | Resampled | Bias | 95% CI | Trend |
| CPL | *Lama glama* | 0.2926 | -0.0253 | 0.2958 | -0.0016 | 0.2817-0.3099 | POS | 0.2930 | -0.0002 | 0.2823-0.3037 | POS |
|  | *Lama guanicoe* | 0.2321 | 0.0352 | 0.2096 | 0.0113 | 0.1534-0.2657 | NEG | 0.2443 | -0.0061 | 0.2352-0.2534 | NEG |
| LN | *Lama glama* | 0.4013 | -0.1340 | 0.4009 | 0.0002 | 0.3583-0.4435 | POS | 0.3972 | 0.0021 | 0.3589-0.4355 | POS |
|  | *Lama guanicoe* | 0.5133 | -0.2460 | 0.4319 | 0.0407 | 0.2883-0.5754 | POS | 0.5488 | -0.0177 | 0.5049-0.5926 | POS |
| HM | *Lama glama* | 0.3011 | -0.0338 | 0.3134 | -0.0061 | 0.2550-0.3718 | ISO | 0.2838 | 0.0087 | 0.2526-0.3150 | ISO |
|  | *Lama guanicoe* | 0.2359 | 0.0314 | 0.1292 | 0.0533 | -0.0467-0.3052 | ISO | 0.2856 | -0.0248 | 0.2634-0.3078 | ISO |
| URP | *Lama glama* | 0.2581 | 0.0092 | 0.2483 | 0.0049 | 0.1851-0.3116 | ISO | 0.2741 | -0.0080 | 0.2365-0.3118 | ISO |
|  | *Lama guanicoe* | 0.1875 | 0.0798 | 0.3874 | -0.0999 | 0.1135-0.6613 | ISO | 0.1644 | 0.0116 | 0.1270-0.2018 | NEG |
| LP | *Lama glama* | 0.3651 | -0.0978 | 0.3585 | 0.0033 | 0.3007-0.4163 | POS | 0.3813 | -0.0081 | 0.3362-0.4264 | POS |
|  | *Lama guanicoe* | 0.2779 | -0.0106 | 0.2465 | 0.0157 | 0.1719-0.3210 | ISO | 0.2933 | -0.0077 | 0.2788-0.3078 | POS |
| BP | *Lama glama* | 0.1633 | 0.1040 | 0.1650 | -0.0009 | 0.1316-0.1985 | NEG | 0.1642 | -0.0004 | 0.1376-0.1907 | NEG |
|  | *Lama guanicoe* | 0.2130 | 0.0543 | 0.0889 | 0.0621 | -0.1108-0.2886 | ISO | 0.2593 | -0.0232 | 0.2332-0.2855 | ISO |
| LO | *Lama glama* | 0.1577 | 0.1096 | 0.1578 | 0.0000 | 0.1404-0.1752 | NEG | 0.1537 | 0.0020 | 0.1386-0.1688 | NEG |
|  | *Lama guanicoe* | 0.0940 | 0.1733 | 0.0943 | -0.0001 | 0.0783-0.1103 | NEG | 0.0949 | -0.0004 | 0.0822-0.1075 | NEG |
| ZB | *Lama glama* | 0.2328 | 0.0345 | 0.2356 | -0.0014 | 0.2194-0.2518 | NEG | 0.2388 | -0.0030 | 0.2282-0.2495 | NEG |
|  | *Lama guanicoe* | 0.1772 | 0.0901 | 0.1463 | 0.0154 | 0.0842-0.2085 | NEG | 0.1929 | -0.0079 | 0.1776-0.2082 | NEG |
| BB | *Lama glama* | 0.0844 | 0.1829 | 0.0907 | -0.0032 | 0.0549-0.1266 | NEG | 0.0765 | 0.0039 | 0.0506-0.1024 | NEG |
|  | *Lama guanicoe* | 0.0397 | 0.2275 | 0.0464 | -0.0033 | 0.0233-0.0695 | NEG | 0.0444 | -0.0023 | 0.0250-0.0638 | NEG |
| HO | *Lama glama* | 0.2523 | 0.0150 | 0.2692 | -0.0084 | 0.1899-0.3484 | ISO | 0.2225 | 0.0149 | 0.1854-0.2597 | NEG |
|  | *Lama guanicoe* | 0.1580 | 0.1093 | 0.2493 | -0.0457 | 0.1146-0.3841 | ISO | 0.1362 | 0.0109 | 0.1147-0.1577 | NEG |
| LD | *Lama glama* | 0.2951 | -0.0278 | 0.2977 | -0.0013 | 0.2829-0.3124 | POS | 0.3017 | -0.0033 | 0.2922-0.3111 | POS |
|  | *Lama guanicoe* | 0.2417 | 0.0256 | 0.2538 | -0.0061 | 0.2331-0.2745 | ISO | 0.2398 | 0.0010 | 0.2298-0.2498 | NEG |
| HD | *Lama glama* | 0.1512 | 0.1161 | 0.1525 | -0.0007 | 0.1235-0.1816 | NEG | 0.1474 | 0.0019 | 0.1242-0.1706 | NEG |
|  | *Lama guanicoe* | 0.2372 | 0.0301 | 0.1719 | 0.0326 | 0.0506-0.2932 | ISO | 0.2619 | -0.0124 | 0.2187-0.3052 | ISO |
| HC | *Lama glama* | 0.3625 | -0.0952 | 0.3659 | -0.0017 | 0.3429-0.3889 | POS | 0.3595 | 0.0015 | 0.3461-0.3729 | POS |
|  | *Lama guanicoe* | 0.4755 | -0.2082 | 0.8473 | -0.1859 | 0.3165-1.3780 | POS | 0.3526 | 0.0615 | 0.3179-0.3872 | POS |
| LPR | *Lama glama* | 0.2119 | 0.0554 | 0.2051 | 0.0034 | 0.1516-0.2585 | NEG | 0.2118 | 0.0000 | 0.1873-0.2364 | NEG |
|  | *Lama guanicoe* | 0.2483 | 0.0190 | 0.3189 | -0.0353 | 0.2324-0.4053 | ISO | 0.2485 | -0.0001 | 0.2215-0.2755 | ISO |

|  |  |  |  | Untrimmed |  |  |  | Trimmed |  |  |  |
| --- | --- | --- | --- | --- | --- | --- | --- | --- | --- | --- | --- |
| Variable | Species | Observed | Departure | Resampled | Bias | 95% CI | Trend | Resampled | Bias | 95% CI | Trend |
| CPL | *Mustela putoriusputorius* | 0.3194 | -0.0521 | 0.3334 | -0.0070 | 0.2924-0.3744 | POS | 0.3139 | 0.0027 | 0.2854-0.3425 | POS |
|  | *Mustela putorius furo* | 0.2836 | -0.0163 | 0.2957 | -0.0061 | 0.2661-0.3253 | ISO | 0.2761 | 0.0037 | 0.2612-0.2910 | ISO |
| LN | *Mustela putoriusputorius* | 0.1559 | 0.1114 | 0.1457 | 0.0051 | 0.0359-0.2555 | NEG | 0.1823 | -0.0132 | 0.1060-0.2585 | NEG |
|  | *Mustela putorius furo* | 0.3646 | -0.0973 | 0.3480 | 0.0083 | 0.2748-0.4211 | POS | 0.3715 | -0.0034 | 0.3385-0.4045 | POS |
| HM | *Mustela putoriusputorius* | 0.2918 | -0.0245 | 0.2751 | 0.0084 | 0.1785-0.3716 | ISO | 0.3045 | -0.0063 | 0.2489-0.3601 | ISO |
|  | *Mustela putorius furo* | 0.2442 | 0.0231 | 0.2390 | 0.0026 | 0.1978-0.2801 | ISO | 0.2505 | -0.0031 | 0.2196-0.2813 | ISO |
| URP | *Mustela putoriusputorius* | 0.4115 | -0.1442 | 0.4369 | -0.0127 | 0.3526-0.5213 | POS | 0.3940 | 0.0088 | 0.3310-0.4570 | POS |
|  | *Mustela putorius furo* | 0.2714 | -0.0041 | 0.2877 | -0.0082 | 0.2332-0.3423 | ISO | 0.2605 | 0.0054 | 0.2227-0.2983 | ISO |
| LP | *Mustela putoriusputorius* | 0.3473 | -0.0800 | 0.3540 | -0.0033 | 0.3232-0.3848 | POS | 0.3460 | 0.0007 | 0.3223-0.3697 | POS |
|  | *Mustela putorius furo* | 0.2992 | -0.0319 | 0.3073 | -0.0041 | 0.2861-0.3284 | POS | 0.3018 | -0.0013 | 0.2859-0.3178 | POS |
| BP | *Mustela putoriusputorius* | 0.1857 | 0.0816 | 0.2048 | -0.0095 | 0.1121-0.2974 | ISO | 0.1639 | 0.0109 | 0.1171-0.2106 | NEG |
|  | *Mustela putorius furo* | 0.2211 | 0.0462 | 0.2226 | -0.0008 | 0.1920-0.2532 | NEG | 0.2217 | -0.0003 | 0.1983-0.2451 | NEG |
| LO | *Mustela putoriusputorius* | 0.1909 | 0.0764 | 0.1956 | -0.0024 | 0.1672-0.2240 | NEG | 0.1944 | -0.0018 | 0.1746-0.2142 | NEG |
|  | *Mustela putorius furo* | 0.2210 | 0.0463 | 0.2153 | 0.0028 | 0.1821-0.2486 | NEG | 0.2290 | -0.0040 | 0.2096-0.2484 | NEG |
| ZB | *Mustela putoriusputorius* | 0.2687 | -0.0014 | 0.2614 | 0.0037 | 0.2211-0.3016 | ISO | 0.2706 | -0.0009 | 0.2377-0.3035 | ISO |
|  | *Mustela putorius furo* | 0.2720 | -0.0047 | 0.2772 | -0.0026 | 0.2575-0.2969 | ISO | 0.2785 | -0.0033 | 0.2629-0.2942 | ISO |
| BB | *Mustela putoriusputorius* | 0.0985 | 0.1688 | 0.0973 | 0.0006 | 0.0340-0.1606 | NEG | 0.1048 | -0.0032 | 0.0524-0.1572 | NEG |
|  | *Mustela putorius furo* | 0.1563 | 0.1110 | 0.1696 | -0.0066 | 0.1275-0.2116 | NEG | 0.1448 | 0.0058 | 0.1246-0.1650 | NEG |
| HO | *Mustela putoriusputorius* | 0.2346 | 0.0327 | 0.2314 | 0.0016 | 0.1949-0.2680 | ISO | 0.2410 | -0.0032 | 0.2121-0.2699 | ISO |
|  | *Mustela putorius furo* | 0.2229 | 0.0444 | 0.2284 | -0.0028 | 0.2063-0.2505 | NEG | 0.2260 | -0.0016 | 0.2096-0.2425 | NEG |
| LD | *Mustela putoriusputorius* | 0.2945 | -0.0272 | 0.3066 | -0.0060 | 0.2673-0.3459 | POS | 0.2925 | 0.0010 | 0.2784-0.3067 | POS |
|  | *Mustela putorius furo* | 0.2641 | 0.0032 | 0.2708 | -0.0033 | 0.2542-0.2874 | ISO | 0.2647 | -0.0003 | 0.2567-0.2727 | ISO |
| HD | *Mustela putoriusputorius* | 0.1204 | 0.1469 | 0.0964 | 0.0120 | 0.0029-0.1899 | NEG | 0.1313 | -0.0054 | 0.0683-0.1942 | NEG |
|  | *Mustela putorius furo* | 0.2541 | 0.0132 | 0.2143 | 0.0199 | 0.0982-0.3305 | ISO | 0.3130 | -0.0295 | 0.2781-0.3478 | POS |
| HC | *Mustela putoriusputorius* | 0.3286 | -0.0613 | 0.3233 | 0.0026 | 0.2899-0.3568 | POS | 0.3367 | -0.0040 | 0.3150-0.3584 | POS |
|  | *Mustela putorius furo* | 0.3187 | -0.0514 | 0.3212 | -0.0013 | 0.3067-0.3357 | POS | 0.3265 | -0.0039 | 0.3161-0.3369 | POS |
| LPR | *Mustela putoriusputorius* | 0.2854 | -0.0181 | 0.2989 | -0.0068 | 0.2558-0.3421 | ISO | 0.2932 | -0.0039 | 0.2561-0.3304 | ISO |
|  | *Mustela putorius furo* | 0.2867 | -0.0194 | 0.3253 | -0.0193 | 0.2191-0.4314 | ISO | 0.2330 | 0.0268 | 0.2161-0.2499 | NEG |

|  |  |  |  | Untrimmed |  |  |  | Trimmed |  |  |  |
| --- | --- | --- | --- | --- | --- | --- | --- | --- | --- | --- | --- |
| Variable | Species | Observed | Departure | Resampled | Bias | 95% CI | Trend | Resampled | Bias | 95% CI | Trend |
| CPL | *Neovisonvison* | 0.2301 | 0.0372 | 0.2309 | -0.0004 | 0.2103-0.2515 | NEG | 0.2295 | 0.0003 | 0.2128-0.2461 | NEG |
|  | *Neovisonvisonletifera* | 0.2172 | 0.0501 | 0.2182 | -0.0005 | 0.1941-0.2423 | NEG | 0.2153 | 0.0010 | 0.1955-0.2350 | NEG |
| LN | *Neovisonvison* | 0.3378 | -0.0705 | 0.3435 | -0.0028 | 0.2346-0.4523 | ISO | 0.3285 | 0.0047 | 0.2730-0.3841 | POS |
|  | *Neovisonvisonletifera* | 0.2884 | -0.0211 | 0.2907 | -0.0012 | 0.2042-0.3772 | ISO | 0.2645 | 0.0119 | 0.2011-0.3280 | ISO |
| HM | *Neovisonvison* | 0.3839 | -0.1166 | 0.3884 | -0.0023 | 0.2891-0.4878 | POS | 0.3954 | -0.0057 | 0.3221-0.4687 | POS |
|  | *Neovisonvisonletifera* | 0.2796 | -0.0123 | 0.2825 | -0.0015 | 0.2426-0.3225 | ISO | 0.2818 | -0.0011 | 0.2455-0.3182 | ISO |
| URP | *Neovisonvison* | 0.2065 | 0.0608 | 0.2053 | 0.0006 | 0.1748-0.2357 | NEG | 0.2094 | -0.0014 | 0.1918-0.2269 | NEG |
|  | *Neovisonvisonletifera* | 0.2181 | 0.0492 | 0.2190 | -0.0005 | 0.1889-0.2492 | NEG | 0.2154 | 0.0013 | 0.1880-0.2427 | NEG |
| LP | *Neovisonvison* | 0.2650 | 0.0023 | 0.2645 | 0.0002 | 0.2175-0.3115 | ISO | 0.2876 | -0.0113 | 0.2610-0.3142 | ISO |
|  | *Neovisonvisonletifera* | 0.2585 | 0.0088 | 0.2600 | -0.0007 | 0.2376-0.2824 | ISO | 0.2584 | 0.0000 | 0.2403-0.2766 | ISO |
| BP | *Neovisonvison* | 0.1364 | 0.1309 | 0.1373 | -0.0005 | 0.1104-0.1642 | NEG | 0.1232 | 0.0066 | 0.1097-0.1368 | NEG |
|  | *Neovisonvisonletifera* | 0.2292 | 0.0381 | 0.2304 | -0.0006 | 0.2006-0.2602 | NEG | 0.2255 | 0.0018 | 0.2002-0.2508 | NEG |
| LO | *Neovisonvison* | 0.1725 | 0.0948 | 0.1764 | -0.0020 | 0.1137-0.2392 | NEG | 0.1510 | 0.0107 | 0.1092-0.1929 | NEG |
|  | *Neovisonvisonletifera* | 0.2077 | 0.0596 | 0.2063 | 0.0007 | 0.1651-0.2476 | NEG | 0.2051 | 0.0013 | 0.1714-0.2388 | NEG |
| ZB | *Neovisonvison* | 0.2680 | -0.0007 | 0.2706 | -0.0013 | 0.2395-0.3017 | ISO | 0.2696 | -0.0008 | 0.2488-0.2903 | ISO |
|  | *Neovisonvisonletifera* | 0.2775 | -0.0102 | 0.2790 | -0.0007 | 0.2572-0.3008 | ISO | 0.2758 | 0.0009 | 0.2568-0.2948 | ISO |
| BB | *Neovisonvison* | 0.1852 | 0.0821 | 0.1952 | -0.0050 | 0.1338-0.2566 | NEG | 0.1955 | -0.0052 | 0.1505-0.2405 | NEG |
|  | *Neovisonvisonletifera* | 0.1978 | 0.0695 | 0.2001 | -0.0012 | 0.1538-0.2464 | NEG | 0.2079 | -0.0050 | 0.1672-0.2485 | NEG |
| HO | *Neovisonvison* | 0.2835 | -0.0162 | 0.2874 | -0.0020 | 0.2470-0.3278 | ISO | 0.2767 | 0.0034 | 0.2478-0.3057 | ISO |
|  | *Neovisonvisonletifera* | 0.2214 | 0.0459 | 0.2234 | -0.0010 | 0.1763-0.2705 | ISO | 0.2155 | 0.0029 | 0.1744-0.2566 | NEG |
| LD | *Neovisonvison* | 0.2766 | -0.0093 | 0.2765 | 0.0001 | 0.2532-0.2997 | ISO | 0.2785 | -0.0009 | 0.2609-0.2960 | ISO |
|  | *Neovisonvisonletifera* | 0.2619 | 0.0054 | 0.2634 | -0.0007 | 0.2391-0.2877 | ISO | 0.2602 | 0.0009 | 0.2388-0.2815 | ISO |
| HD | *Neovisonvison* | 0.3687 | -0.1014 | 0.3752 | -0.0033 | 0.3273-0.4232 | POS | 0.3628 | 0.0030 | 0.3377-0.3878 | POS |
|  | *Neovisonvisonletifera* | 0.4242 | -0.1569 | 0.4271 | -0.0014 | 0.3758-0.4784 | POS | 0.4255 | -0.0006 | 0.3785-0.4724 | POS |
| HC | *Neovisonvison* | 0.2793 | -0.0120 | 0.2766 | 0.0014 | 0.2263-0.3268 | ISO | 0.2754 | 0.0020 | 0.2355-0.3154 | ISO |
|  | *Neovisonvisonletifera* | 0.3316 | -0.0643 | 0.3334 | -0.0009 | 0.3093-0.3575 | POS | 0.3319 | -0.0002 | 0.3104-0.3535 | POS |
| LPR | *Neovisonvison* | 0.2177 | 0.0496 | 0.2162 | 0.0007 | 0.1827-0.2497 | NEG | 0.2132 | 0.0022 | 0.1861-0.2403 | NEG |
|  | *Neovisonvisonletifera* | 0.2405 | 0.0268 | 0.2407 | -0.0001 | 0.2069-0.2746 | ISO | 0.2493 | -0.0044 | 0.2240-0.2745 | ISO |

|  |  |  |  | Untrimmed |  |  |  | Trimmed |  |  |  |
| --- | --- | --- | --- | --- | --- | --- | --- | --- | --- | --- | --- |
| Variable | Species | Observed | Departure | Resampled | Bias | 95% CI | Trend | Resampled | Bias | 95% CI | Trend |
| CPL | *Oryctolaguscuniculus f. domesticus* | 0.2917 | -0.0244 | 0.2818 | 0.0050 | 0.2614-0.3021 | ISO | 0.2954 | -0.0018 | 0.2888-0.3019 | POS |
|  | *Oryctolaguscuniculus* | 0.2767 | -0.0094 | 0.2753 | 0.0007 | 0.2626-0.2879 | ISO | 0.2765 | 0.0001 | 0.2678-0.2853 | POS |
| LN | *Oryctolaguscuniculus f. domesticus* | 0.3540 | -0.0867 | 0.3501 | 0.0020 | 0.3336-0.3665 | POS | 0.3524 | 0.0008 | 0.3401-0.3647 | POS |
|  | *Oryctolaguscuniculus* | 0.3905 | -0.1232 | 0.3894 | 0.0005 | 0.3633-0.4155 | POS | 0.3957 | -0.0026 | 0.3811-0.4104 | POS |
| HM | *Oryctolaguscuniculus f. domesticus* | 0.2831 | -0.0158 | 0.2929 | -0.0049 | 0.2743-0.3114 | POS | 0.2856 | -0.0012 | 0.2770-0.2942 | POS |
|  | *Oryctolaguscuniculus* | 0.2513 | 0.0160 | 0.2514 | 0.0000 | 0.2385-0.2642 | NEG | 0.2501 | 0.0006 | 0.2394-0.2607 | NEG |
| URP | *Oryctolaguscuniculus f. domesticus* | 0.1869 | 0.0804 | 0.1536 | 0.0167 | 0.0937-0.2134 | NEG | 0.2065 | -0.0098 | 0.1956-0.2174 | NEG |
|  | *Oryctolaguscuniculus* | 0.2356 | 0.0317 | 0.2379 | -0.0012 | 0.2097-0.2661 | NEG | 0.2293 | 0.0032 | 0.2160-0.2426 | NEG |
| LP | *Oryctolaguscuniculus f. domesticus* | 0.3400 | -0.0727 | 0.3176 | 0.0112 | 0.2759-0.3593 | POS | 0.3535 | -0.0068 | 0.3461-0.3609 | POS |
|  | *Oryctolaguscuniculus* | 0.3009 | -0.0336 | 0.2977 | 0.0016 | 0.2831-0.3123 | POS | 0.3025 | -0.0008 | 0.2915-0.3134 | POS |
| BP | *Oryctolaguscuniculus f. domesticus* | 0.2561 | 0.0112 | 0.2806 | -0.0122 | 0.2370-0.3241 | ISO | 0.2493 | 0.0034 | 0.2354-0.2632 | NEG |
|  | *Oryctolaguscuniculus* | 0.1980 | 0.0693 | 0.1912 | 0.0034 | 0.1633-0.2192 | NEG | 0.2150 | -0.0085 | 0.2058-0.2242 | NEG |
| LO | *Oryctolaguscuniculus f. domesticus* | 0.2325 | 0.0348 | 0.1937 | 0.0194 | 0.1235-0.2638 | NEG | 0.2536 | -0.0106 | 0.2416-0.2657 | NEG |
|  | *Oryctolaguscuniculus* | 0.2416 | 0.0257 | 0.2430 | -0.0007 | 0.2319-0.2540 | NEG | 0.2411 | 0.0002 | 0.2323-0.2499 | NEG |
| ZB | *Oryctolaguscuniculus f. domesticus* | 0.2005 | 0.0668 | 0.2188 | -0.0091 | 0.1865-0.2510 | NEG | 0.1946 | 0.0030 | 0.1856-0.2035 | NEG |
|  | *Oryctolaguscuniculus* | 0.1964 | 0.0709 | 0.1981 | -0.0009 | 0.1871-0.2092 | NEG | 0.1928 | 0.0018 | 0.1867-0.1990 | NEG |
| BB | *Oryctolaguscuniculus f. domesticus* | 0.0981 | 0.1692 | 0.1212 | -0.0115 | 0.0799-0.1624 | NEG | 0.0889 | 0.0046 | 0.0767-0.1011 | NEG |
|  | *Oryctolaguscuniculus* | 0.1196 | 0.1477 | 0.1230 | -0.0017 | 0.0980-0.1480 | NEG | 0.1092 | 0.0052 | 0.0964-0.1220 | NEG |
| HO | *Oryctolaguscuniculus f. domesticus* | 0.1894 | 0.0779 | 0.1561 | 0.0167 | 0.0964-0.2157 | NEG | 0.2083 | -0.0095 | 0.2003-0.2164 | NEG |
|  | *Oryctolaguscuniculus* | 0.2176 | 0.0497 | 0.2222 | -0.0023 | 0.1870-0.2575 | NEG | 0.2191 | -0.0008 | 0.2110-0.2273 | NEG |
| LD | *Oryctolaguscuniculus f. domesticus* | 0.3012 | -0.0339 | 0.2816 | 0.0098 | 0.2417-0.3216 | ISO | 0.3069 | -0.0029 | 0.2929-0.3209 | POS |
|  | *Oryctolaguscuniculus* | 0.2859 | -0.0186 | 0.2830 | 0.0015 | 0.2694-0.2966 | POS | 0.2869 | -0.0005 | 0.2778-0.2961 | POS |
| HD | *Oryctolaguscuniculus f. domesticus* | 0.3110 | -0.0437 | 0.3638 | -0.0264 | 0.2709-0.4568 | POS | 0.2943 | 0.0083 | 0.2816-0.3070 | POS |
|  | *Oryctolaguscuniculus* | 0.3180 | -0.0507 | 0.3207 | -0.0014 | 0.3082-0.3332 | POS | 0.3195 | -0.0007 | 0.3097-0.3293 | POS |
| HC | *Oryctolaguscuniculus f. domesticus* | 0.3529 | -0.0856 | 0.3950 | -0.0211 | 0.3239-0.4661 | POS | 0.3308 | 0.0110 | 0.3219-0.3396 | POS |
|  | *Oryctolaguscuniculus* | 0.3629 | -0.0956 | 0.3667 | -0.0019 | 0.3515-0.3819 | POS | 0.3622 | 0.0004 | 0.3529-0.3715 | POS |
| LPR | *Oryctolaguscuniculus f. domesticus* | 0.2042 | 0.0631 | 0.2110 | -0.0034 | 0.1982-0.2238 | NEG | 0.2039 | 0.0001 | 0.1971-0.2107 | NEG |
|  | *Oryctolaguscuniculus* | 0.2223 | 0.0450 | 0.2246 | -0.0011 | 0.2095-0.2396 | NEG | 0.2239 | -0.0008 | 0.2152-0.2326 | NEG |

|  |  |  |  | Untrimmed |  |  |  | Trimmed |  |  |  |
| --- | --- | --- | --- | --- | --- | --- | --- | --- | --- | --- | --- |
| Variable | Species | Observed | Departure | Resampled | Bias | 95% CI | Trend | Resampled | Bias | 95% CI | Trend |
| CPL | *Ovisaries* | 0.2811 | -0.0138 | 0.2843 | -0.0016 | 0.2718-0.2968 | POS | 0.2808 | 0.0002 | 0.2715-0.2901 | POS |
|  | *Ovismusimon* | 0.2693 | -0.0020 | 0.2700 | -0.0004 | 0.2529-0.2871 | ISO | 0.2751 | -0.0029 | 0.2686-0.2816 | POS |
| LN | *Ovisaries* | 0.3584 | -0.0911 | 0.3610 | -0.0013 | 0.3287-0.3933 | POS | 0.3589 | -0.0003 | 0.3335-0.3843 | POS |
|  | *Ovismusimon* | 0.3758 | -0.1085 | 0.3792 | -0.0017 | 0.3392-0.4191 | POS | 0.3570 | 0.0094 | 0.3379-0.3760 | POS |
| HM | *Ovisaries* | 0.3465 | -0.0792 | 0.3426 | 0.0020 | 0.2979-0.3872 | POS | 0.3474 | -0.0004 | 0.3124-0.3823 | POS |
|  | *Ovismusimon* | 0.3793 | -0.1120 | 0.3957 | -0.0082 | 0.2948-0.4966 | POS | 0.3315 | 0.0239 | 0.2906-0.3724 | POS |
| URP | *Ovisaries* | 0.2825 | -0.0152 | 0.2773 | 0.0026 | 0.2458-0.3088 | ISO | 0.2863 | -0.0019 | 0.2604-0.3123 | ISO |
|  | *Ovismusimon* | 0.2666 | 0.0007 | 0.2614 | 0.0026 | 0.2228-0.2999 | ISO | 0.2761 | -0.0048 | 0.2583-0.2939 | ISO |
| LP | *Ovisaries* | 0.3172 | -0.0499 | 0.3204 | -0.0016 | 0.3051-0.3357 | POS | 0.3140 | 0.0016 | 0.3036-0.3245 | POS |
|  | *Ovismusimon* | 0.2697 | -0.0024 | 0.2673 | 0.0012 | 0.2264-0.3082 | ISO | 0.2870 | -0.0087 | 0.2745-0.2995 | POS |
| BP | *Ovisaries* | 0.2241 | 0.0432 | 0.2275 | -0.0017 | 0.1987-0.2563 | NEG | 0.2160 | 0.0041 | 0.1980-0.2340 | NEG |
|  | *Ovismusimon* | 0.2393 | 0.0280 | 0.2556 | -0.0082 | 0.1713-0.3399 | ISO | 0.2039 | 0.0177 | 0.1764-0.2314 | NEG |
| LO | *Ovisaries* | 0.1561 | 0.1112 | 0.1606 | -0.0022 | 0.1376-0.1835 | NEG | 0.1612 | -0.0026 | 0.1451-0.1773 | NEG |
|  | *Ovismusimon* | 0.1658 | 0.1015 | 0.1680 | -0.0011 | 0.1531-0.1829 | NEG | 0.1638 | 0.0010 | 0.1550-0.1725 | NEG |
| ZB | *Ovisaries* | 0.2371 | 0.0302 | 0.2355 | 0.0008 | 0.2085-0.2625 | NEG | 0.2407 | -0.0018 | 0.2236-0.2577 | NEG |
|  | *Ovismusimon* | 0.2101 | 0.0572 | 0.2124 | -0.0012 | 0.1951-0.2297 | NEG | 0.2027 | 0.0037 | 0.1938-0.2116 | NEG |
| BB | *Ovisaries* | 0.1262 | 0.1411 | 0.1158 | 0.0052 | 0.0510-0.1807 | NEG | 0.1068 | 0.0097 | 0.0643-0.1494 | NEG |
|  | *Ovismusimon* | 0.1612 | 0.1061 | 0.1518 | 0.0047 | 0.0670-0.2366 | NEG | 0.1566 | 0.0023 | 0.1031-0.2101 | NEG |
| HO | *Ovisaries* | 0.1947 | 0.0726 | 0.2017 | -0.0035 | 0.1681-0.2354 | NEG | 0.1961 | -0.0007 | 0.1679-0.2242 | NEG |
|  | *Ovismusimon* | 0.1692 | 0.0981 | 0.1614 | 0.0039 | 0.0924-0.2305 | NEG | 0.2067 | -0.0188 | 0.1838-0.2297 | NEG |
| LD | *Ovisaries* | 0.2795 | -0.0122 | 0.2790 | 0.0003 | 0.2606-0.2974 | ISO | 0.2860 | -0.0032 | 0.2767-0.2952 | POS |
|  | *Ovismusimon* | 0.2633 | 0.0040 | 0.2631 | 0.0001 | 0.2405-0.2857 | ISO | 0.2735 | -0.0051 | 0.2632-0.2837 | ISO |
| HD | *Ovisaries* | 0.1480 | 0.1193 | 0.1485 | -0.0002 | 0.1199-0.1770 | NEG | 0.1419 | 0.0030 | 0.1230-0.1609 | NEG |
|  | *Ovismusimon* | 0.1646 | 0.1027 | 0.1623 | 0.0011 | 0.1141-0.2105 | NEG | 0.1582 | 0.0032 | 0.1235-0.1929 | NEG |
| HC | *Ovisaries* | 0.3303 | -0.0630 | 0.3405 | -0.0051 | 0.3007-0.3804 | POS | 0.3215 | 0.0044 | 0.2947-0.3484 | POS |
|  | *Ovismusimon* | 0.3393 | -0.0720 | 0.3436 | -0.0022 | 0.3122-0.3750 | POS | 0.3340 | 0.0027 | 0.3159-0.3521 | POS |
| LPR | *Ovisaries* | 0.3136 | -0.0463 | 0.3114 | 0.0011 | 0.2807-0.3421 | POS | 0.3184 | -0.0024 | 0.2944-0.3425 | POS |
|  | *Ovismusimon* | 0.3218 | -0.0545 | 0.3252 | -0.0017 | 0.2821-0.3683 | POS | 0.3174 | 0.0022 | 0.2885-0.3463 | POS |

|  |  |  |  | Untrimmed |  |  |  | Trimmed |  |  |  |
| --- | --- | --- | --- | --- | --- | --- | --- | --- | --- | --- | --- |
| Variable | Species | Observed | Departure | Resampled | Bias | 95% CI | Trend | Resampled | Bias | 95% CI | Trend |
| CPL | *Sus scrofa domestica* | 0.2828 | -0.0155 | 0.2842 | -0.0007 | 0.2633-0.3051 | ISO | 0.2752 | 0.0038 | 0.2643-0.2862 | ISO |
|  | *Sus scrofascrofa* | 0.2845 | -0.0172 | 0.2853 | -0.0004 | 0.2772-0.2934 | POS | 0.2839 | 0.0003 | 0.2771-0.2906 | POS |
| LN | *Sus scrofa domestica* | 0.3472 | -0.0799 | 0.3471 | 0.0000 | 0.3239-0.3703 | POS | 0.3489 | -0.0009 | 0.3310-0.3668 | POS |
|  | *Sus scrofascrofa* | 0.3684 | -0.1011 | 0.3691 | -0.0004 | 0.3517-0.3865 | POS | 0.3735 | -0.0025 | 0.3611-0.3859 | POS |
| HM | *Sus scrofa domestica* | 0.2171 | 0.0502 | 0.2157 | 0.0007 | 0.1925-0.2390 | NEG | 0.2235 | -0.0032 | 0.2059-0.2411 | NEG |
|  | *Sus scrofascrofa* | 0.2332 | 0.0341 | 0.2321 | 0.0005 | 0.2132-0.2510 | NEG | 0.2362 | -0.0015 | 0.2236-0.2488 | NEG |
| URP | *Sus scrofa domestica* | 0.3275 | -0.0602 | 0.3301 | -0.0013 | 0.2833-0.3769 | POS | 0.3205 | 0.0035 | 0.2934-0.3477 | POS |
|  | *Sus scrofascrofa* | 0.3242 | -0.0569 | 0.3269 | -0.0013 | 0.2956-0.3582 | POS | 0.3129 | 0.0057 | 0.2919-0.3339 | POS |
| LP | *Sus scrofa domestica* | 0.3247 | -0.0574 | 0.3254 | -0.0003 | 0.3006-0.3502 | POS | 0.3221 | 0.0013 | 0.3102-0.3341 | POS |
|  | *Sus scrofascrofa* | 0.3145 | -0.0472 | 0.3148 | -0.0001 | 0.3047-0.3250 | POS | 0.3142 | 0.0002 | 0.3074-0.3211 | POS |
| BP | *Sus scrofa domestica* | 0.1629 | 0.1044 | 0.1593 | 0.0018 | 0.1263-0.1922 | NEG | 0.1635 | -0.0003 | 0.1376-0.1895 | NEG |
|  | *Sus scrofascrofa* | 0.1735 | 0.0938 | 0.1740 | -0.0003 | 0.1495-0.1985 | NEG | 0.1752 | -0.0009 | 0.1541-0.1964 | NEG |
| LO | *Sus scrofa domestica* | 0.1221 | 0.1452 | 0.1222 | -0.0001 | 0.1096-0.1348 | NEG | 0.1229 | -0.0004 | 0.1131-0.1327 | NEG |
|  | *Sus scrofascrofa* | 0.1241 | 0.1432 | 0.1240 | 0.0001 | 0.1115-0.1364 | NEG | 0.1249 | -0.0004 | 0.1140-0.1358 | NEG |
| ZB | *Sus scrofa domestica* | 0.2214 | 0.0459 | 0.2221 | -0.0003 | 0.2079-0.2363 | NEG | 0.2182 | 0.0016 | 0.2076-0.2288 | NEG |
|  | *Sus scrofascrofa* | 0.2104 | 0.0569 | 0.2109 | -0.0003 | 0.1979-0.2239 | NEG | 0.2084 | 0.0010 | 0.1986-0.2182 | NEG |
| BB | *Sus scrofa domestica* | 0.0967 | 0.1706 | 0.0967 | 0.0000 | 0.0860-0.1075 | NEG | 0.0964 | 0.0002 | 0.0869-0.1059 | NEG |
|  | *Sus scrofascrofa* | 0.0923 | 0.1750 | 0.0925 | -0.0001 | 0.0804-0.1047 | NEG | 0.0953 | -0.0015 | 0.0860-0.1045 | NEG |
| HO | *Sus scrofa domestica* | 0.2465 | 0.0208 | 0.2457 | 0.0004 | 0.2341-0.2574 | NEG | 0.2506 | -0.0021 | 0.2427-0.2585 | NEG |
|  | *Sus scrofascrofa* | 0.2453 | 0.0220 | 0.2452 | 0.0001 | 0.2363-0.2541 | NEG | 0.2447 | 0.0003 | 0.2369-0.2525 | NEG |
| LD | *Sus scrofa domestica* | 0.2770 | -0.0097 | 0.2767 | 0.0001 | 0.2655-0.2879 | ISO | 0.2790 | -0.0010 | 0.2725-0.2856 | POS |
|  | *Sus scrofascrofa* | 0.2980 | -0.0307 | 0.2982 | -0.0001 | 0.2872-0.3092 | POS | 0.2999 | -0.0010 | 0.2911-0.3087 | POS |
| HD | *Sus scrofa domestica* | 0.2836 | -0.0163 | 0.2835 | 0.0001 | 0.2668-0.3001 | ISO | 0.2800 | 0.0018 | 0.2663-0.2937 | ISO |
|  | *Sus scrofascrofa* | 0.2907 | -0.0234 | 0.2908 | 0.0000 | 0.2741-0.3074 | POS | 0.2890 | 0.0009 | 0.2761-0.3018 | POS |
| HC | *Sus scrofa domestica* | 0.3122 | -0.0449 | 0.3152 | -0.0015 | 0.2969-0.3336 | POS | 0.3040 | 0.0041 | 0.2936-0.3144 | POS |
|  | *Sus scrofascrofa* | 0.3048 | -0.0375 | 0.3057 | -0.0005 | 0.2893-0.3222 | POS | 0.3039 | 0.0004 | 0.2906-0.3172 | POS |
| LPR | *Sus scrofa domestica* | 0.3528 | -0.0855 | 0.3543 | -0.0007 | 0.3231-0.3855 | POS | 0.3611 | -0.0041 | 0.3443-0.3779 | POS |
|  | *Sus scrofascrofa* | 0.3166 | -0.0493 | 0.3146 | 0.0010 | 0.2835-0.3457 | POS | 0.3222 | -0.0028 | 0.2959-0.3484 | POS |
