# Supplementary material for: On the lack of a universal pattern associated with mammalian domestication: differences in skull growth trajectories across phylogeny
Source: R Soc Open Sci. 2017 Oct 25;4(10):170876. doi: 10.1098/rsos.170876 (PMC5666271; doi:10.1098/rsos.170876)
Supplement: Supplementary Information 3 [file rsos170876supp3.docx]

**Supplementary Information 3**.Test for common slopes and common intercepts of bivariate regressions for the domesticated and wild forms in the species investigated. b1common, common slope from reduced major axis analysis; log(b0)common, common intercept from reduced major axis analysis; Lr, likelihood ratio (Warton et al., 2006); *w*, Wald statistic (Warton et al., 2006); Pb1, *p*-value of Lr parameter; P(logb0), *p*-value of *w* parameter. Associated p < 0.05. Bonferroni correction for all comparisons p=0.0036, except fpr *Capra hircus*-*C. aegargus* comparison, where p=0.005. Asterisks indicate non-significant differences in slopes or intercepts under Bomferroni correction.Abbreviations as in Fig. 2.

| Variable | Slope |  |  | Intercept |  |  | Shift |
| --- | --- | --- | --- | --- | --- | --- | --- |
|  | *Lr* | *p* b_1_ | b_1_ common | *W* log b_0_ | *p*log b_0_ | Log b_0_ common |  |
| CPL | 2.9136 | 0.0878 | 1.1826 | 0.3236 | 0.5694 | 0.3075 |  |
| HM | 1.2758 | 0.2587 | 1.2799 | 2.4356 | 0.1186 | -0.4520 |  |
| URP | 0.9454 | 0.3309 | 1.1022 | 0.4563 | 0.4993 | -0.1176 |  |
| LP | 0.1782 | 0.6729 | 1.3311 | 0.0084 | 0.9270 | -0.1048 |  |
| BP | 0.0935 | 0.7598 | 1.0218 | 4.2548 | 0.0391 | D > W |  |
| LO | 0.0541 | 0.8161 | 0.6135 | 1.1841 | 0.2765 | 0.08147 |  |
| ZB | 0.0522 | 0.8192 | 1.2029 | 0.1927 | 0.6606 | -0.0407 |  |
| BB | 0.6286 | 0.4279 | 0.4643 | 0.2392 | 0.6247 | 0.5184 |  |
| HO | 0.2179 | 0.6406 | 1.0232 | 0.0181 | 0.8928 | -0.1437 |  |
| LD | 1.5227 | 0.2172 | 1.1479 | 0.0714 | 0.7893 | 0.2806 |  |
| HD | 0.0869 | 0.7682 | 1.0633 | 1.4023 | 0.2363 | -0.4817 |  |
| HC | 0.0065 | 0.9355 | 1.5893 | 0.0079 | 0.9291 | -0.5085 |  |
| LPR | 3.0569 | 0.0804 | 1.3385 | 0.7607 | 0.3831 | -0.4119 |  |

Test for common slopes and common intercepts of bivariate regressions for *Camelusbactrianus*&*Camelusferus*

| Variable | Slope |  |  | Intercept |  |  | Shift |
| --- | --- | --- | --- | --- | --- | --- | --- |
|  | *Lr* | *p* b_1_ | b_1_ common | *W* log b_0_ | *p*log b_0_ | Log b_0_ common |  |
| CPL | 9.3933 | 0.0022 | D > W |  |  |  |  |
| LN | 0.0814 | 0.7754 | 1.5460 | 51.7168 | 0.0000 | D > W |  |
| HM | 1.3718 | 0.2416 | 1.0135 | 18.3514 | 0.0000 | D > W |  |
| URP | 0.8637 | 0.3527 | 1.4556 | 1.9540 | 0.1621 | -0.3786 | W > D |
| LP | 11.0610 | 0.0009 | D > W |  |  |  |  |
| BP | 1.9995 | 0.1573 | 0.8373 | 1.3765 | 0.24069 | 0.0608 | W > D |
| LO | 0.3890 | 0.5328 | 0.7821 | 42.4234 | 0.0000 | D > W |  |
| ZB | 14.3338 | 0.0001 | W > D |  |  |  |  |
| BB | 0.1087 | 0.7415 | 0.4484 | 1.6054 | 0.2051 | 0.3903 | W > D |
| HO | 9.1272 | 0.0025 | W > D |  |  |  |  |
| LD | 0.2262 | 0.6343 | 1.1859 | 16.3129 | 0.0000 | D > W |  |
| HD | 1.3595 | 0.2436 | 1.0520 | 20.0557 | 0.0000 | W > D |  |
| HC | 3.0257 | 0.0819 | 1.3841 | 17.2123 | 0.0000 | D > W |  |
| LPR | 24.9490 | 0.0000 | D > W |  |  |  |  |

Test for common slopes and common intercepts of bivariate regressions for *Canis lupus familiaris*&*Canis lupus familiaris*

| Variable | Slope |  |  | Intercept |  |  | Shift |
| --- | --- | --- | --- | --- | --- | --- | --- |
|  | *Lr* | *p* b_1_ | b_1_ common | *W* log b_0_ | *p*log b_0_ | Log b_0_ common |  |
| CPL | 2.7242 | 0.0988 | 1.1309 | 19.3602 | 0.0000 | W > D |  |
| LN | 8.8697 | 0.0029 | W > D |  |  |  |  |
| HM | 1.7057 | 0.1915 | 1.2919 | 9.3846 | 0.0022 | W > D |  |
| URP | 0.1129 | 0.7369 | 1.1187 | 0.4230 | 0.5154 | -0.1412 | D > W |
| LP | 0.9823 | 0.3216 | 1.2539 | 7.2772 | 0.0070* | W > D |  |
| BP | 1.3206 | 0.2505 | 0.9701 | 4.5922 | 0.0321* | W > D |  |
| LO | 1.5277 | 0.2165 | 0.6432 | 9.1799 | 0.0024 | D > W |  |
| ZB | 0.0032 | 0.9550 | 0.9232 | 0.9042 | 0.3417 | 0.2204 | D > W |
| BB | 1.8797 | 0.1704 | 0.5811 | 0.5028 | 0.4783 | 0.3668 | D > W |
| HO | 12.8939 | 0.0003 | D > W | 19.3602 | 0.0000 | W > D |  |

Test for common slopes and common intercepts of bivariate regressions for *Capra hircus*& *Capra aegagrus*

| Variable | Slope |  |  | Intercept |  |  | Shift |
| --- | --- | --- | --- | --- | --- | --- | --- |
|  | *Lr* | *p* b_1_ | b_1_ common | *W* log b_0_ | *p* log b_0_ | Log b_0_ common |  |
| CPL | 4.2690 | 0.0388 | W > D |  |  |  |  |
| LN | 13.0778 | 0.0003 | D > W |  |  |  |  |
| HM | 1.4592 | 0.2270 | 1.0589 | 2.3907 | 0.1221 | -0.0713 |  |
| URP | 0.4585 | 0.4983 | 0.9763 | 0.3190 | 0.5722 | -0.0976 |  |
| LP | 0.2901 | 0.5901 | 1.2501 | 0.5513 | 0.4578 | 0.0949 |  |
| BP | 6.3037 | 0.0120* | W > D |  |  |  |  |
| LO | 0.0000 | 0.9972 | 0.8945 | 0.0392 | 0.8430 | -0.1340 |  |
| ZB | 0.1049 | 0.7460 | 1.0783 | 0.0794 | 0.7781 | 0.2363 |  |
| BB | 2.3893 | 0.1222 | 0.5469 | 20.0610 | 0.0000 | D > W |  |
| HO | 0.1677 | 0.6821 | 0.7542 | 22.6255 | 0.0000 | D > W |  |
| LD | 1.4505 | 0.2284 | 1.0686 | 0.7432 | 0.3886 | 0.3868 |  |
| HD | 3.0386 | 0.0813 | 1.3323 | 2.8192 | 0.0931 | -0.3848 |  |
| HC | 4.3861 | 0.0362* | W > D |  |  |  |  |
| LPR | 6.6495 | 0.0099* | W > D |  |  |  |  |

Test for common slopes and common intercepts of bivariate regressions for *Caviaporcellus*&*Caviaaperea*

| Variable | Slope |  |  | Intercept |  |  | Shift |
| --- | --- | --- | --- | --- | --- | --- | --- |
|  | *Lr* | *p* b_1_ | b_1_ common | *W* log b_0_ | *p* log b_0_ | Log b_0_ common |  |
| CPL | 3.5457 | 0.0597 | 1.1281 | 4.0401 | 0.0444* | D > W |  |
| LN | 0.0188 | 0.8909 | 1.4634 | 0.5030 | 0.4781 | -0.3405 | D > W |
| HM | 0.3409 | 0.5593 | 1.4791 | 9.7513 | 0.0018 | W > D |  |
| URP | 1.7518 | 0.1856 | 1.0975 | 7.5663 | 0.0059* | W > D |  |
| LP | 0.0607 | 0.8054 | 1.2838 | 0.2582 | 0.6113 | -0.0786 | D > W |
| BP | 1.5462 | 0.2137 | 1.0331 | 0.8503 | 0.3565 | -0.3556 | D > W |
| LO | 5.6721 | 0.0172* | D > W |  |  |  |  |
| ZB | 16.6049 | 0.0000 | W > D |  |  |  |  |
| BB | 4.5931 | 0.0321* | D > W |  |  |  |  |
| HO | 7.5128 | 0.0061* | D > W |  |  |  |  |
| LD | 0.0356 | 0.8503 | 1.1473 | 0.0083 | 0.9275 | 0.2805 | D > W |
| HD | 0.0466 | 0.8290 | 1.2195 | 68.6908 | 0.0000 | W > D |  |
| HC | 4.4060 | 0.0358* | W > D |  |  |  |  |
| LPR | 2.2155 | 0.1366 | 1.1428 | 6.2071 | 0.0127* | W > D |  |

Test for common slopes and common intercepts of bivariate regressions for *Equusferuscaballus*&*Equusferusprzewalskii*

| Variable | Slope |  |  | Intercept |  |  | Shift |
| --- | --- | --- | --- | --- | --- | --- | --- |
|  | *Lr* | *p* b_1_ | b_1_ common | *W* log b_0_ | *p* log b_0_ | Log b_0_ common |  |
| CPL | 1.6783 | 0.1951 | 1.0884 | 0.8020 | 0.3705 | 0.3599 | W > D |
| LN | 3.4074 | 0.0649 | 1.2386 | 0.1000 | 0.7517 | -0.3256 | W > D |
| HM | 8.1403 | 0.0043* | W > D |  |  |  |  |
| URP | 1.8056 | 0.1790 | 1.0669 | 19.5288 | 0.0000 | W > D |  |
| LP | 1.2064 | 0.2720 | 1.1019 | 0.4576 | 0.4988 | -0.0299 | W > D |
| BP | 0.5470 | 0.4595 | 1.2669 | 1.4440 | 0.2295 | -0.1382 | W > D |
| LO | 0.7541 | 0.3852 | 1.1041 | 0.5971 | 0.4397 | -0.1421 | W > D |
| ZB | 0.1888 | 0.6639 | 1.1551 | 14.7519 | 0.0001 | D > W |  |
| BB | 22.4102 | 0.0000 | W > D |  |  |  |  |
| HO | 0.8293 | 0.3624 | 0.8234 | 8.7381 | 0.0031 | D > W |  |
| LD | 2.2302 | 0.1353 | 1.2009 | 6.3484 | 0.0117* | D > W |  |
| HD | 6.1787 | 0.0129* | W > D |  |  |  |  |
| HC | 3.8282 | 0.0504 | 1.4892 | 11.5485 | 0.0007 | D > W |  |
| LPR | 5.4757 | 0.0193* | W > D |  |  |  |  |

Test for common slopes and common intercepts of bivariate regressions for *Felissilvestriscatus*&*Felissilvestrislybica*

| Variable | Slope |  |  | Intercept |  |  | Shift |
| --- | --- | --- | --- | --- | --- | --- | --- |
|  | *Lr* | *p* b_1_ | b_1_ common | *W* log b_0_ | *p* log b_0_ | Log b_0_ common |  |
| CPL | 6.8155 | 0.0090* | W > D |  |  |  |  |
| LN | 2.2784 | 0.1312 | 2.8482 | 58.1805 | 0.0000 | W > D |  |
| HM | 0.1712 | 0.6790 | 1.1872 | 15.1799 | 0.0000 | D > W |  |
| URP | 0.8337 | 0.3612 | 1.3601 | 3.5306 | 0.0602 | -0.2823 |  |
| LP | 14.9873 | 0.0001 | W > D |  |  |  |  |
| BP | 0.0154 | 0.9012 | 0.8724 | 4.7948 | 0.0285* | D > W |  |
| LO | 5.5300 | 0.0187* | D > W |  |  |  |  |
| ZB | 0.9439 | 0.3313 | 0.8100 | 66.0139 | 0.0000 | D > W |  |
| BB | 5.4631 | 0.0194* | D > W |  |  |  |  |
| HO | 4.3729 | 0.0365* | W > D |  |  |  |  |
| LD | 6.0018 | 0.0143* | W > D |  |  |  |  |
| HD | 0.5167 | 0.4722 | 1.0543 | 3.8549 | 0.0496* | D > W |  |
| HC | 17.1346 | 0.0000 | W > D |  |  |  |  |
| LPR | 7.5015 | 0.0062* | W > D |  |  |  |  |

Test for common slopes and common intercepts of bivariate regressions for *Lama pacos*&*Vicugnavicugna*

| Variable | Slope |  |  | Intercept |  |  | Shift |
| --- | --- | --- | --- | --- | --- | --- | --- |
|  | *Lr* | *p* b_1_ | b_1_ common | *W* log b_0_ | *p* log b_0_ | Log b_0_ common |  |
| CPL | 0.1559 | 0.6930 | 1.1643 | 0.0003 | 0.9855 | 0.4150 | W > D |
| LN | 17.0604 | 0.0000 | W > D |  |  |  |  |
| HM | 0.0401 | 0.8413 | 1.2320 | 18.3431 | 0.0000 | D > W |  |
| URP | 1.5443 | 0.2140 | 1.1654 | 0.2664 | 0.6058 | -0.1447 | W > D |
| LP | 1.4015 | 0.2365 | 1.4198 | 7.6641 | 0.0056* | W > D |  |
| BP | 13.6938 | 0.0002 | W > D |  |  |  |  |
| LO | 1.1753 | 0.2783 | 0.6291 | 1.0625 | 0.3026 | 0.1123 | W > D |
| ZB | 0.0109 | 0.9168 | 0.9330 | 19.9936 | 0.0000 | D > W |  |
| BB | 0.1258 | 0.7228 | 0.4094 | 3.5544 | 0.0594 | 0.4691 | W > D |
| HO | 3.0767 | 0.0794 | 0.9935 | 10.2612 | 0.0013 | D > W |  |
| LD | 0.0008 | 0.9778 | 1.1793 | 0.3834 | 0.5358 | 0.3251 | W > D |
| HD | 16.2743 | 0.0000 | W > D |  |  |  |  |
| HC | 16.0885 | 0.0000 | W > D |  |  |  |  |
| LPR | 7.3942 | 0.0065* | W > D |  |  |  |  |

Test for common slopes and common intercepts of bivariate regressions for *Lama glama*& *Lama guanicoe*

| Variable | Slope |  |  | Intercept |  |  | Shift |
| --- | --- | --- | --- | --- | --- | --- | --- |
|  | *Lr* | *p* b_1_ | b_1_ common | *W* log b_0_ | *p* log b_0_ | Log b_0_ common |  |
| CPL | 5.4302 | 0.0198* | D > W |  |  |  |  |
| LN | 0.0120 | 0.9128 | 1.6151 | 0.9557 | 0.3283 | -0.4490 |  |
| HM | 3.5774 | 0.0586 | 1.2916 | 0.4610 | 0.4971 | -0.3073 | W > D |
| URP | 27.3018 | 0.0000 | D > W |  |  |  |  |
| LP | 6.1828 | 0.0129* | D > W |  |  |  |  |
| BP | 0.1390 | 0.7093 | 1.0068 | 3.9504 | 0.0469* | W > D |  |
| LO | 1.2338 | 0.2667 | 0.9199 | 17.9868 | 0.0000 | D > W |  |
| ZB | 0.3068 | 0.5797 | 1.1270 | 0.3304 | 0.5654 | 0.2266 |  |
| BB | 0.5842 | 0.4446 | 0.8587 | 3.3649 | 0.0666 | 0.1734 | W > D |
| HO | 2.3477 | 0.1255 | 0.9793 | 0.8541 | 0.3554 | -0.0452 | W > D |
| LD | 4.5614 | 0.0327* | D > W |  |  |  |  |
| HD | 0.1676 | 0.6822 | 1.1905 | 3.5384 | 0.0599 | -0.5067 |  |
| HC | 0.8644 | 0.3525 | 1.3020 | 1.0148 | 0.3138 | -0.1354 |  |
| LPR | 0.3511 | 0.5535 | 1.2221 | 2.8713 | 0.0902 | -0.0892 |  |

Test for common slopes and common intercepts of bivariate regressions for *Mustelaputoriusputorius*&*Mustelaputoriusfuro*

| Variable | Slope |  |  | Intercept |  |  | Shift |
| --- | --- | --- | --- | --- | --- | --- | --- |
|  | *Lr* | *p* b_1_ | b_1_ common | *W* log b_0_ | *p* log b_0_ | Log b_0_ common |  |
| CPL | 0.0676 | 0.7949 | 0.9508 | 26.8866 | 0.0000 | D > W |  |
| LN | 1.2320 | 0.2670 | 1.5951 | 12.5439 | 0.0004 | W > D |  |
| HM | 2.1143 | 0.1459 | 1.4621 | 0.7470 | 0.3874 | -0.4185 |  |
| URP | 3.6784 | 0.0551 | 0.9882 | 5.8951 | 0.0152* | D > W |  |
| LP | 0.0163 | 0.8983 | 1.1159 | 14.4326 | 0.0001 | D > W |  |
| BP | 18.3887 | 0.0000 | W > D |  |  |  |  |
| LO | 3.0834 | 0.0791 | 0.9964 | 1.8323 | 0.1758 | -0.2953 |  |
| ZB | 2.6213 | 0.1054 | 1.1708 | 1.3936 | 0.2378 | 0.2138 |  |
| BB | 1.8547 | 0.1732 | 1.0079 | 4.6137 | 0.03172* | W > D |  |
| HO | 0.0265 | 0.8707 | 1.1698 | 10.1174 | 0.0014 | D > W |  |
| LD | 0.0714 | 0.7892 | 1.1529 | 42.7453 | 0.0000 | D > W |  |
| HD | 6.4905 | 0.0108* | W > D |  |  |  |  |
| HC | 3.4149 | 0.0646 | 1.3964 | 0.2712 | 0.6025 | -0.1684 |  |
| LPR | 3.5737 | 0.0587 | 1.0742 | 3.7674 | 0.0523 | -0.0287 |  |

Test for common slopes and common intercepts of bivariate regressions for *Neovison vison* &*Neovison vison letifera*

| Variable | Slope |  |  | Intercept |  |  | Shift |
| --- | --- | --- | --- | --- | --- | --- | --- |
|  | *Lr* | *p* b_1_ | b_1_ common | *W* log b_0_ | *p* log b_0_ | Log b_0_ common |  |
| CPL | 1.4522 | 0.2282 | 1.1835 | 2.5633 | 0.1094 | 0.3661 | D > W |
| LN | 9.8872 | 0.0017 | W > D |  |  |  |  |
| HM | 3.0641 | 0.0800 | 1.1326 | 36.1662 | 0.0000 | D > W |  |
| URP | 19.4909 | 0.0000 | W > D |  |  |  |  |
| LP | 14.9291 | 0.0001 | D > W |  |  |  |  |
| BP | 8.1293 | 0.0043* | D > W |  |  |  |  |
| LO | 1.0085 | 0.3153 | 1.0175 | 66.5412 | 0.0000 | W > D |  |
| ZB | 0.0496 | 0.8237 | 0.8281 | 1.3371 | 0.2475 | 0.2601 | D > W |
| BB | 5.5154 | 0.0188* | W > D |  |  |  |  |
| HO | 10.2977 | 0.0013 | W > D |  |  |  |  |
| LD | 1.5063 | 0.2197 | 1.1971 | 44.0238 | 0.0000 | W > D |  |
| HD | 0.2329 | 0.6294 | 1.3283 | 23.9022 | 0.0000 | D > W |  |
| HC | 0.9821 | 0.3217 | 1.4983 |  |  |  |  |
| LPR | 6.7766 | 0.0092* | W > D |  |  |  |  |

Test for common slopes and common intercepts of bivariate regressions for *Oryctolaguscuniculus f. domesticus*&*Oryctolaguscuniculus*

| Variable | Slope |  |  | Intercept |  |  | Shift |
| --- | --- | --- | --- | --- | --- | --- | --- |
|  | *Lr* | *p* b_1_ | b_1_ common | *W* log b_0_ | *p* log b_0_ | Log b_0_ common |  |
| CPL | 1.6226 | 0.2027 | 1.0899 | 0.2156 | 0.6424 | 0.4017 | D > W |
| LN | 0.4883 | 0.4847 | 1.5001 | 0.8832 | 0.3473 | -0.3607 | D > W |
| HM | 0.7646 | 0.3819 | 1.5313 | 12.1691 | 0.0005 | D > W |  |
| URP | 0.9501 | 0.3297 | 1.1703 | 0.1871 | 0.6653 | -0.1760 | D > W |
| LP | 10.0359 | 0.0015 | D > W |  |  |  |  |
| BP | 0.6624 | 0.4157 | 0.9960 | 0.9981 | 0.3177 | -0.2387 | D > W |
| LO | 0.0002 | 0.9873 | 0.6733 | 46.0433 | 0.0000 | W > D |  |
| ZB | 6.8176 | 0.0090* | D > W |  |  |  |  |
| BB | 0.1277 | 0.7208 | 0.8762 | 9.7620 | 0.0018 | W > D |  |
| HO | 1.2690 | 0.2599 | 0.8291 | 1.1612 | 0.2812 | -0.0199 | D > W |
| LD | 1.5921 | 0.2070 | 1.0834 | 0.0605 | 0.8057 | 0.2945 | D > W |
| HD | 0.1407 | 0.7076 | 0.7527 | 11.6140 | 0.0006 | D > W |  |
| HC | 0.1319 | 0.7165 | 1.3607 | 3.5980 | 0.0578 | -0.2008 | D > W |
| LPR | 0.0015 | 0.9688 | 1.3266 | 0.6105 | 0.4346 | -0.2881 | D > W |

Test for common slopes and common intercepts of bivariate regressions for *Ovisaries*&*Ovismusimon*

| Variable | Slope |  |  | Intercept |  |  | Shift |
| --- | --- | --- | --- | --- | --- | --- | --- |
|  | *Lr* | *p* b_1_ | b_1_ common | *W* log b_0_ | *p* log b_0_ | Log b_0_ common |  |
| CPL | 0.0000 | 0.9996 | 1.2140 | 4.1728 | 0.0411* | W > D |  |
| LN | 2.6042 | 0.1066 | 1.5553 | 8.2524 | 0.0041* | W > D |  |
| HM | 0.7072 | 0.4004 | 0.9862 | 3.8431 | 0.0499* | D > W |  |
| URP | 0.2441 | 0.6213 | 1.4175 | 2.4792 | 0.1154 | -0.3803 |  |
| LP | 2.8443 | 0.0917 | 1.3456 | 6.3408 | 0.0118* | W > D |  |
| BP | 0.0056 | 0.9402 | 0.7919 | 14.5604 | 0.0001 | D > W |  |
| LO | 0.1669 | 0.6829 | 0.5550 | 2.8656 | 0.0905 | -0.0029 |  |
| ZB | 1.2442 | 0.2647 | 0.9361 | 65.9003 | 0.0000 | D > W |  |
| BB | 0.0938 | 0.7594 | 0.4292 | 55.2832 | 0.0000 | W > D |  |
| HO | 0.2614 | 0.6092 | 1.0497 | 15.1017 | 0.0001 | D > W |  |
| LD | 10.9728 | 0.0009 | W > D |  |  |  |  |
| HD | 0.0828 | 0.7736 | 1.2335 | 29.7153 | 0.0000 | D > W |  |
| HC | 1.1135 | 0.2913 | 1.3602 | 24.4185 | 0.0000 | D > W |  |
| LPR | 2.5252 | 0.1120 | 1.4799 | 0.02699 | 0.8695 | -0.4192 |  |

Test for common slopes and common intercepts of bivariate regressions for *Susscrofadomestica*&*Susscrofascrofa*
