# Supplementary material for: On the lack of a universal pattern associated with mammalian domestication: differences in skull growth trajectories across phylogeny
Source: R Soc Open Sci. 2017 Oct 25;4(10):170876. doi: 10.1098/rsos.170876 (PMC5666271; doi:10.1098/rsos.170876)
Supplement: Supplementary Information 4 [file rsos170876supp4.docx]

**Supplementary Information 4**. Bivariate plots of ontogenetic trajectories of Log transformed cranial variables versus the geometric mean for 13 comparisons. Black dots and regression correspond to domestic form. Red dots and regression correspond to wild forms. Abbreviations as in Fig. 2.

Bivariate plots of ontogenetic trajectories of Log transformed cranial variables versus the geometric mean for *Camelus bactrianus & Camelus ferus*

Bivariate plots of ontogenetic trajectories of Log transformed cranial variables versus the geometric mean for *Canis lupus familiaris & Canis lupus familiaris*

Bivariate plots of ontogenetic trajectories of Log transformed cranial variables versus the geometric mean for *Capra hircus & Capra aegagrus*

Bivariate plots of ontogenetic trajectories of Log transformed cranial variables versus the geometric mean for *Cavia porcellus & Cavia aperea*

Bivariate plots of ontogenetic trajectories of Log transformed cranial variables versus the geometric mean for *Equus ferus caballus & Equus ferus przewalskii*

Bivariate plots of ontogenetic trajectories of Log transformed cranial variables versus the geometric mean for *Felis silvestris catus & Felis silvestris lybica*

Bivariate plots of ontogenetic trajectories of Log transformed cranial variables versus the geometric mean for *Lama pacos & Vicugna vicugna*

Bivariate plots of ontogenetic trajectories of Log transformed cranial variables versus the geometric mean for *Lama glama & Lama guanicoe*

Bivariate plots of ontogenetic trajectories of Log transformed cranial variables versus the geometric mean for *Mustela putorius putorius & Mustela putorius furo*

Bivariate plots of ontogenetic trajectories of Log transformed cranial variables versus the geometric mean for *Neovison vison & Neovison vison letifera*

Bivariate plots of ontogenetic trajectories of Log transformed cranial variables versus the geometric mean for *Oryctolagus cuniculus f. domesticus & Oryctolagus cuniculus*

Bivariate plots of ontogenetic trajectories of Log transformed cranial variables versus the geometric mean for *Ovis aries & Ovis musimon*

Bivariate plots of ontogenetic trajectories of Log transformed cranial variables versus the geometric mean for *Sus scrofa domestica & Sus scrofa scrofa*
