# Supplementary material for: On the lack of a universal pattern associated with mammalian domestication: differences in skull growth trajectories across phylogeny
Source: R Soc Open Sci. 2017 Oct 25;4(10):170876. doi: 10.1098/rsos.170876 (PMC5666271; doi:10.1098/rsos.170876)
Supplement: Supplementary Information 5 [file rsos170876supp5.docx]

**Supplementary Information 5**. Adjusted coefficient of correlation of cranial variables with the geometric mean of domestic and wild forms. Abbreviations as in Fig. 2.

|  | CPL | LN | HM | URP | LP | BP | LO | ZB | BB | HO | LD | HD | HC | LPR |
| --- | --- | --- | --- | --- | --- | --- | --- | --- | --- | --- | --- | --- | --- | --- |
| *Canis lupus familiaris* | 0.9877 | 0.9620 | 0.9415 | 0.9140 | 0.9803 | 0.8976 | 0.9583 | 0.9518 | 0.7045 | 0.9016 | 0.9872 | 0.8957 | 0.9727 | 0.8217 |
| *Canis lupus lupus* | 0.9942 | 0.9741 | 0.8204 | 0.9012 | 0.9837 | 0.9377 | 0.9353 | 0.9342 | 0.0014 | 0.9126 | 0.9894 | 0.8557 | 0.9867 | 0.8969 |
| *Mustela putorius furo* | 0.7863 | 0.2574 | 0.6385 | 0.7677 | 0.8164 | 0.5992 | 0.7698 | 0.8283 | 0.2640 | 0.7747 | 0.8700 | 0.1557 | 0.8561 | 0.7250 |
| *Mustela putorius putorius* | 0.9358 | 0.7554 | 0.6646 | 0.8722 | 0.9180 | 0.7376 | 0.8078 | 0.9154 | 0.5771 | 0.8747 | 0.9450 | 0.6249 | 0.9409 | 0.8571 |
| *Neovison vison* | 0.9663 | 0.8827 | 0.8557 | 0.9141 | 0.9244 | 0.9026 | 0.7868 | 0.9666 | 0.7783 | 0.9586 | 0.9424 | 0.9485 | 0.8906 | 0.8834 |
| *Neovison vison letifera* | 0.8947 | 0.5202 | 0.7066 | 0.6667 | 0.9251 | 0.8194 | 0.6885 | 0.8684 | 0.5631 | 0.6542 | 0.9007 | 0.7420 | 0.8946 | 0.6857 |
| *Felis silvestris catus* | 0.9831 | 0.9424 | 0.7418 | 0.8893 | 0.9801 | 0.9102 | 0.9674 | 0.9837 | 0.6272 | 0.9324 | 0.9775 | 0.9177 | 0.9582 | 0.9035 |
| *Felis silvestris lybica* | 0.9267 | 0.6832 | 0.4558 | 0.7559 | 0.7985 | 0.8765 | 0.8130 | 0.9382 | 0.2507 | 0.6592 | 0.8461 | 0.6824 | 0.8584 | 0.7799 |
| *Equus ferus caballus* | 0.9534 | 0.8756 | 0.9307 | 0.8015 | 0.9574 | 0.8219 | 0.7015 | 0.9413 | 0.3670 | 0.7887 | 0.9478 | 0.5733 | 0.8809 | 0.7327 |
| *Equus ferus przewalskii* | 0.9924 | 0.9593 | 0.9788 | 0.9588 | 0.9906 | 0.9660 | 0.8669 | 0.9831 | 0.7027 | 0.8542 | 0.9872 | 0.9645 | 0.9871 | 0.9466 |
| *Capra hircus* | 0.9863 | 0.9507 | 0.9396 | 0.9026 | 0.9775 | 0.8227 | 0.9043 | 0.9381 | 0.6983 | 0.8584 | ? | ? | ? | ? |
| *Capra aegagrus* | 0.9770 | 0.6759 | 0.8645 | 0.8932 | 0.9514 | 0.8808 | 0.9786 | 0.9623 | 0.5839 | 0.7741 | ? | ? | ? | ? |
| *Ovis aries* | 0.9731 | 0.9172 | 0.8531 | 0.7962 | 0.9587 | 0.8569 | 0.8516 | 0.9293 | 0.4078 | 0.8177 | 0.9593 | 0.6394 | 0.9095 | 0.8185 |
| *Ovis musimon* | 0.9904 | 0.9556 | 0.8806 | 0.8813 | 0.9691 | 0.8477 | 0.9611 | 0.9683 | 0.5567 | 0.7880 | 0.9749 | 0.6845 | 0.9424 | 0.9058 |
| *Sus scrofa domestica* | 0.9784 | 0.9683 | 0.9369 | 0.9252 | 0.9757 | 0.7828 | 0.9052 | 0.9777 | 0.9059 | 0.9841 | 0.9907 | 0.9668 | 0.9774 | 0.9458 |
| *Sus scrofa scrofa* | 0.9919 | 0.9836 | 0.9585 | 0.9485 | 0.9906 | 0.8212 | 0.8585 | 0.9786 | 0.8977 | 0.9902 | 0.9895 | 0.9745 | 0.9522 | 0.8991 |
| *Lama glama* | 0.9898 | 0.9437 | 0.9339 | 0.8728 | 0.9155 | 0.8182 | 0.9432 | 0.9880 | 0.7352 | 0.8936 | 0.9876 | 0.7903 | 0.9723 | 0.8664 |
| *Lama guanicoe* | 0.9295 | 0.8346 | 0.8766 | 0.5687 | 0.9225 | 0.6346 | 0.7029 | 0.8593 | 0.1655 | 0.6792 | 0.9054 | 0.6397 | 0.7752 | 0.8185 |
| *Lama pacos* | 0.9459 | 0.8554 | 0.6266 | 0.7735 | 0.9590 | 0.3905 | 0.8376 | 0.9363 | 0.3723 | 0.6317 | 0.8672 | 0.3580 | 0.8802 | 0.8345 |
| *Vicugna vicugna* | 0.9595 | 0.8150 | 0.8128 | 0.7080 | 0.8976 | 0.1610 | 0.7378 | 0.8799 | 0.1301 | 0.6529 | 0.9197 | 0.4663 | 0.9028 | 0.6854 |
| *Camelus bactrianus* | 0.9626 | ? | 0.9881 | 0.9785 | 0.9916 | 0.9933 | 0.9363 | 0.9742 | 0.9283 | 0.9929 | 0.9931 | 0.9577 | 0.9667 | 0.9672 |
| *Camelus ferus* | 0.9919 | ? | 0.9604 | 0.9093 | 0.9644 | 0.9191 | 0.9591 | 0.9900 | 0.7570 | 0.9736 | 0.9781 | 0.8087 | 0.9660 | 0.9173 |
| *Oryctolagus cuniculus domesticus* | 0.9916 | 0.9869 | 0.9815 | 0.9639 | 0.9901 | 0.9548 | 0.9591 | 0.9691 | 0.8214 | 0.9661 | 0.9633 | 0.9471 | 0.9751 | 0.9775 |
| *Oryctolagus cuniculus* | 0.9595 | 0.9478 | 0.9090 | 0.9254 | 0.9739 | 0.8430 | 0.9233 | 0.9608 | 0.7538 | 0.8945 | 0.9743 | 0.9352 | 0.9440 | 0.9158 |
| *Cavia porcellus* | 0.8412 | 0.8367 | 0.9301 | 0.8661 | 0.9412 | 0.8203 | 0.6940 | 0.8835 | 0.2401 | 0.7020 | 0.8882 | 0.6852 | 0.8212 | 0.7889 |
| *Cavia aperea* | 0.9573 | 0.8518 | 0.9377 | 0.8999 | 0.9451 | 0.6349 | 0.7908 | 0.9461 | 0.7567 | 0.8092 | 0.8765 | 0.3229 | 0.7550 | 0.8690 |
