# Supplementary material for: On the lack of a universal pattern associated with mammalian domestication: differences in skull growth trajectories across phylogeny
Source: R Soc Open Sci. 2017 Oct 25;4(10):170876. doi: 10.1098/rsos.170876 (PMC5666271; doi:10.1098/rsos.170876)
Supplement: Supplementary Information 6 [file rsos170876supp6.docx]

**Supplementary Information 6**. Summary of results of the bivariate analysis of ontogenetic trajectories in the 13 wild and domesticated forms and 14 skull variables investigated. The symbols indicate the cases in which domesticates (D) were larger than wild (W) forms (or vice-versa) out of the 14 relations between size (with geometric mean as a proxy for it) and the variable in question. For those trajectories that exhibit the same slopes and intercepts, we evaluated which form, if any, exhibits significant extensions of the trajectory with respect to the other (‘shift’). Abbreviations as in figure 2. See summary of this Table in Fig. 3.

|  | *Canis lupus familiaris/ Canis lupus lupus* | | | *Mustela putorius furo/ Mustela putorius putorius* | | | *Neovison vison/ Neovison vison letifera* | | | *Felis silvestris catus/ Felis silvestris lybica* | | |
| --- | --- | --- | --- | --- | --- | --- | --- | --- | --- | --- | --- | --- |
| Variables | Slope | Intercept | Shift | Slope | Intercept | Shift | Slope | Intercept | Shift | Slope | Intercept | Shift |
| CPL | D > W |  |  | D > W |  |  |  | D > W |  |  |  | W > D |
| LN |  | D > W |  |  |  |  |  | W > D |  |  |  | W > D |
| HM |  | D > W |  |  |  | W > D |  |  |  | W > D |  |  |
| URP |  |  | W > D | D > W |  |  |  | D > W |  |  | W > D |  |
| LP | D > W |  |  | D > W |  |  |  | D > W |  |  |  | W > D |
| BP |  |  | W > D |  | W > D |  | W > D |  |  |  |  | W > D |
| LO |  | D > W |  |  | D > W |  |  |  |  |  |  | W > D |
| ZB | W > D |  |  |  |  |  |  |  |  |  | D > W |  |
| BB |  |  | W > D |  |  | W > D |  | W > D |  | W > D |  |  |
| HO | W > D |  |  |  |  | W > D |  | D > W |  |  | D > W |  |
| LD |  | D > W |  | D > W |  |  |  | D > W |  |  | D > W |  |
| HD | W > D |  |  |  |  |  | W > D |  |  | W > D |  |  |
| HC |  | D > W |  |  |  |  |  |  |  |  | D > W |  |
| LPR | D > W |  |  |  |  |  |  |  |  | W > D |  |  |
| D > W | 3(14) Acceleration | 5(9) Post-displacement | 0(3) | 4(14)  Acceleration | 1(10) Post-displacement | 0(3) | 0(14) | 5(12) Post-displacement | 0(0) | 0(14) | 4(10) Post-displacement | 0(5) |
| W > D | 3(14)  Deceleration | 0(9) | 3(3) Hypomorphosis | 0(14) | 1(10) Pre-displacement | 3(3) Hypomorphosis | 2(14) Deceleration | 2(12) Pre-displacement | 0(0) | 4(14) Deceleration | 1(10) Pre-displacement | 5(5)  Hypomorphosis |

|  | *Equus ferus caballus/ Equus ferus przewalskii* | | | *Capra hircus/ Capra aegagrus* | | | *Ovis aries/ Ovis musimon* | | | *Sus scrofa domestica/ Sus scrofa scrofa* | | |
| --- | --- | --- | --- | --- | --- | --- | --- | --- | --- | --- | --- | --- |
| Variables | Slope | Intercept | Shift | Slope | Intercept | Shift | Slope | Intercept | Shift | Slope | Intercept | Shift |
| CPL |  | D > W |  |  | W > D |  |  |  | D > W |  | W > D |  |
| LN |  |  | D > W | W > D |  |  |  |  | D > W |  | W > D |  |
| HM |  | W > D |  |  | W > D |  |  | D > W |  |  | D > W |  |
| URP |  | W > D |  |  |  | D > W |  |  | D > W |  |  |  |
| LP |  |  | D > W |  | W > D |  | D > W |  |  |  | W > D |  |
| BP |  |  | D > W |  | W > D |  |  |  | D > W |  | D > W |  |
| LO | D > W |  |  |  | D > W |  |  | W > D |  |  |  |  |
| ZB | W > D |  |  |  |  | D > W | D > W |  |  |  | D > W |  |
| BB | D > W |  |  |  |  | D > W |  | W > D |  |  | W > D |  |
| HO | D > W |  |  | D > W |  |  |  |  | D > W |  | D > W |  |
| LD |  |  | D > W | ? | ? | ? |  |  | D > W | W > D |  |  |
| HD |  | W > D |  | ? | ? | ? |  | D > W |  |  | D > W |  |
| HC | W > D |  |  | ? | ? | ? |  |  | D > W |  | D > W |  |
| LPR |  | W > D |  | ? | ? | ? |  |  | D > W |  |  |  |
| D > W | 3(14) Acceleration | 1(9) Post-displacement | 4(4) Hypermorphosis | 1(10) Acceleration | 1(8) Post-displacement | 3(3) Hypermorphosis | 2(14) Acceleration | 2(12) Post-displacement | 8(8)  Hypermorphosis | 0(14) | 6(13) Post-displacement | 0(0) |
| W > D | 2(14) Deceleration | 4(9) Pre-displacement | 0(4) | 1(10) Deceleration | 4(8) Pre-displacement | 0(3) | 0(14) | 2(12) Pre-displacement | 0(8) | 1(14) Deceleration | 4(13) Pre-displacement | 0(0) |

|  | *Lama glama/ Lama guanicoe* | | | *Lama pacos/ Vicugna vicugna* | | | *Camelus bactrianus/ Camelus ferus* | | | *Oryctolagus cuniculus f. domesticus/ Oryctolagus cuniculus* | | |
| --- | --- | --- | --- | --- | --- | --- | --- | --- | --- | --- | --- | --- |
| Variables | Slope | Intercept | Shift | Slope | Intercept | Shift | Slope | Intercept | Shift | Slope | Intercept | Shift |
| CPL |  |  | W > D | W > D |  |  |  |  |  |  |  | D > W |
| LN | W > D |  |  |  | W > D |  | ? | ? | ? | W > D |  |  |
| HM |  | D > W |  |  | D > W |  |  |  |  |  | D > W |  |
| URP |  |  | W > D |  |  |  |  |  |  | W > D |  |  |
| LP |  | W > D |  | W > D |  |  |  |  |  | D > W |  |  |
| BP | W > D |  |  |  | D > W |  |  | D > W |  | D > W |  |  |
| LO |  |  | W > D | D > W |  |  |  |  |  |  | W > D |  |
| ZB |  | D > W |  |  | D > W |  |  |  |  |  |  | D > W |
| BB |  |  | W > D | D > W |  |  |  |  |  | W > D |  |  |
| HO |  | D > W |  | W > D |  |  |  |  |  | W > D |  |  |
| LD |  |  | W > D | W > D |  |  |  |  |  |  | W > D |  |
| HD | W > D |  |  |  | D > W |  |  |  |  |  | D > W |  |
| HC | W > D |  |  | W > D |  |  |  |  |  |  | W > D |  |
| LPR | W > D |  |  | W > D |  |  |  |  |  | W > D |  |  |
| D > W | 0(14) | 3(9) Post-displacement | 0(5) | 2(14) Acceleration | 4(6) Post-displacement | 0(0) | 0(13) | 1(13) Post-displacement | 0(0) | 2(14) Acceleration | 2(7) Post-displacement | 2(2) Hypermorphosis |
| W > D | 5(14) Deceleration | 1(9) Pre-displacement | 5(5) Hypomorphosis | 6(14) Deceleration | 1(6) Pre-displacement | 0(0) | 0(13) | 0(13) | 0(0) | 5(14) Deceleration | 3(7) Pre-displacement | 0(2) |

|  | *Cavia porcellus/ Cavia aperea* | | |
| --- | --- | --- | --- |
| Variables | Slope | Intercept | Shift |
| CPL | W > D |  |  |
| LN | D > W |  |  |
| HM |  |  |  |
| URP |  |  |  |
| LP |  |  |  |
| BP | W > D |  |  |
| LO |  |  |  |
| ZB |  |  |  |
| BB |  | D > W |  |
| HO |  | D > W |  |
| LD |  |  |  |
| HD |  |  |  |
| HC | W > D |  |  |
| LPR | W > D |  |  |
| D > W | 1(14) Acceleration | 2(9) Post-displacement | 0(0) |
| W > D | 4(14) Deceleration | 0(9) | 0(0) |
